# Identification of a novel GREMLIN1 uptake pathway in epithelial cells that requires BMP binding

**DOI:** 10.1016/j.jbc.2025.110780

**Published:** 2025-09-29

**Authors:** Zhichun Gao, Yuhan Gao, Louise R. Dutton, Melibea Berzosa Suner, Grace Todd, Gregory R. Gipson, Connor Brown, Emma M. Kerr, Carole Daly, Bianca Plouffe, Philip D. Dunne, Dessislava Malinova, Derek P. Brazil

**Affiliations:** 1 Wellcome-Wolfson Institute for Experimental Medicine, School of Medicine, Dentistry, and Biomedical Sciences, Queen’s University Belfast, Belfast, Northern Ireland, UK; 2 Institute of Cancer Sciences, Wolfson Wohl Cancer Research Centre, University of Glasgow, Glasgow, Scotland, UK; 3 Cardiovascular Research Institute, Massachusetts General Hospital, Harvard Medical School, Boston, Massachusetts, USA; 4 Patrick G. Johnston Center for Cancer Research, School of Medicine, Dentistry, and Biomedical Sciences, Queen’s University Belfast, Belfast, Northern Ireland, UK

**Keywords:** Gremlin1 (GREM1), bone morphogenetic protein (BMP), epithelium, endocytosis, plasma membrane, cell signaling

## Abstract

GREM1 binding to BMP targets in the extracellular matrix prevents their engagement with cognate BMP receptors, attenuating BMP-dependent signaling and gene expression. Some evidence suggests that GREM1 can directly bind to receptor tyrosine kinases on the plasma membrane, further complicating our understanding of GREM1 biology. To attempt to clarify the complexities of GREM1 signaling, we show that GREM1 protein is produced and secreted by intestinal fibroblasts and endocytosed by neighboring epithelial cells. GREM1 uptake occurs by both clathrin- and caveolin-mediated endocytosis. Cell membrane heparin sulfate proteoglycans are required for GREM1 binding and uptake, and once internalized, GREM1 appears to localize to the early endosomes and can be resecreted. The addition of BMP2 enhanced GREM1 uptake into cells. Remarkably, the generation of a BMP-resistant GREM1 mutant abolished GREM1 uptake both in the presence and in the absence of BMP2. These data suggest that GREM1 binding and uptake into cells requires BMP binding, a process that may contribute to the antagonism of BMP signaling by GREM1.

Gremlin1 (GREM1) is a secreted glycoprotein antagonist of bone morphogenetic proteins. GREM1 binds to BMP targets such as BMP2 and BMP4 to sequester them in the extracellular matrix, preventing BMP-mediated receptor binding and activation (1). Exquisite temporospatial control of GREM1 and BMP expression is required for normal mammalian limb and kidney development. GREM1 is the BMP antagonist required for maintenance of the apical ectodermal ridge (AER) *via* regulation of sonic hedgehog (Shh) and fibroblast growth factor (Fgf) signaling during limb development (2, 3, 4).

Mice homozygous for *Grem1* gene deletion display defective fore and hindlimb formation, as well as renal agenesis, leading to death shortly after birth (2, 3). These defects are thought to be due to inappropriately amplified BMP4 signaling, as deletion of a single allele of Bmp4 can rescue ureteric bud outgrowth and kidney morphogenesis in *Grem1*−/− mice (5). GREM1 expression in adult tissues has been identified mainly in stromal fibroblasts. For example, GREM1 expression in a distinct population of fibroblasts was shown to play a key role in intestinal regeneration after injury (6). GREM1 expression has also been described in a range of stem cells, including intestinal mesenchymal stem cells (7), osteochondroreticular cells (8), and glioma cancer stem cells (9).

Along with its critical role in development, GREM1 upregulation has been reported in a range of human pathologies. Elevated GREM1 mRNA in samples from patients with idiopathic pulmonary fibrosis (10, 11), diabetic nephropathy (12), chronic pancreatitis (13) and osteoarthritis (14). GREM1 overexpression has also been detailed in a wide range of human cancers, including colorectal (15, 16), mesothelioma (17), gastric (18, 19), breast (20, 21), and glioma (9, 22). Interactions between GREM1^+^ cancer-associated fibroblasts (CAFs) and SPP/osteopontin^+^ macrophages were associated with poorer patient outcomes in gastric cancer (23). A rare inherited condition called hereditary mixed polyposis syndrome (HMPS) is caused by a 40 kb chromosomal duplication event on chromosome 15q13.3 upstream of the GREM1 gene that leads to 2500-fold upregulation of GREM1 mRNA production in the intestine of these patients (24). A mouse model of HMPS with aberrant GREM1 overexpression in intestinal epithelial cells recapitulated this proliferative phenotype, suggesting that high levels of GREM1 alone can drive intestinal cell growth, intestinal polyp, and tumor formation (16). Importantly, treatment with an anti-Grem1 antibody abrogated this pro-cancer polyposis phenotype (25).

Most data in the literature suggest that GREM1 is a “bad actor” in human cancer, with high levels of GREM1 associated with worse patient outcomes (*e.g.* (16, 26)). However, a small number of papers argue the opposite and suggest that high levels of GREM1 are prognostic of less aggressive tumor phenotypes and improved patient outcomes in both pancreatic adenocarcinoma (27) and colorectal cancer (28) (reviewed in (29)). In addition to its canonical role as a secreted ligand-trap antagonist for BMP2 and other BMPs, some reports have suggested a non-canonical, direct signaling capacity for GREM1. GREM1 was reported to bind and activate the vascular endothelial growth factor receptor-2 (VEGFR2), initiating angiogenesis (30, 31). However, other groups, including our own, reported conflicting data in this regard (26, 32). GREM1 was reported to bind to the fibroblast growth factor receptor 1 (FGFR1), activating MEK/ERK signaling and driving castration-resistant prostate cancer growth (33). Together with other reports that GREM1 can activate epidermal growth factor (EGF) receptor in breast cancer cells (20, 34) and bind to slit guidance ligand-2 (SLIT2) in neurons, a somewhat muddied picture exists of GREM1 biology and signaling in health and disease. To address some of the gaps in our understanding of GREM1 biology, we have interrogated the membrane binding, secretion, and uptake of GREM1 into mammalian cells. We report that GREM1 is secreted, binds to, and is endocytosed in colorectal cancer cells, a process that requires BMP binding.

## Results

**Figure 1:**
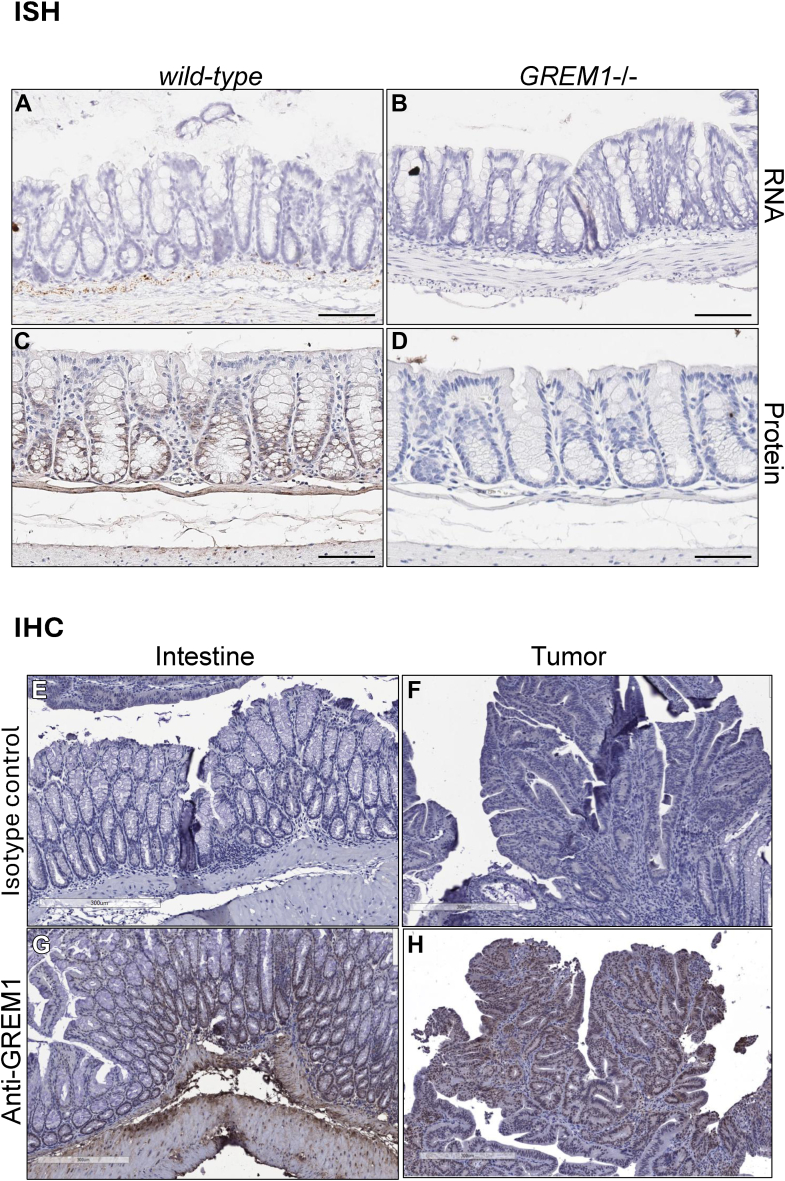
**Differential pattern of GREM1 mRNA and protein expression in healthy and colorectal cancer intestine.** FFPE colon sections (5 μm) from *wild-type* or *GREM1*−/− mice were analyzed for GREM1 mRNA and protein expression. *In situ* hybridization (*A*, *B*) and immunohistochemistry (*C*, *D*) were performed as described in Methods. FFPE colon sections (5 μm) from *Apc*^*fl/fl*^*;Kras*^*Lsl-G12D*^*;Tp53*^*fl/fl*^*;villin-CreERT2* (AKP) mice were stained for GREM1 protein (*E*-*H*) as described in Methods. Images from both mouse intestine (*E*, *G*) and tumor (*F*, *H*) were captured after staining with goat IgG isotype control (*E*, *F*) or anti-GREM1 antibody (*G*, *H*). Positive GREM1 protein staining was visualized using DAB (*brown*), and sections were counterstained with hemotoxylin (*blue*) and imaged using PathXL and Aperio ImageScope. Scale bars 100 μm (*A*-*D*), or 300 μm (*E*-*H*). Images are representative of staining from n = 4 mice.

*In situ* hybridization (ISH) of wild-type and *GREM1*−/− mouse colon FFPE sections identified a staining pattern consistent with GREM1 mRNA localization to the muscularis layer and submucosa (Fig. 1*A*). No *GREM1* mRNA was detected in the colonic crypts, epithelial or other cells (Fig. 1*A*). The specificity of the ISH probes was confirmed by the absence of any positive signal in FFPE sections from *GREM1*−/− mice (Fig. 1*B*). In contrast, GREM1 protein was more widely detected in the mouse colon, with staining detected in epithelial cells at the base of the crypts, as well as in the muscularis and submucosa layers (Fig. 1*C*). Concerns around the specificity of the anti-GREM1 antibody used for immunohistochemistry staining were allayed by the absence of any GREM1 protein signal in colon tissue from *GREM1*−/− mice (Fig. 1*D*). These results are consistent with previous data from our group characterizing GREM1 mRNA/protein localization in the mouse intestine (26). To extend these findings to a colorectal cancer model, colon sections from *Apc*^*fl/fl*^*;Kras*^*Lsl-G12D*^*;Tp53*^*fl/fl*^*;villin-CreERT2* mice (35, 36, 37) were probed for GREM1 protein expression. GREM1 protein expression was detected in the muscularis and submucosa layers of the colon, as well as in intestinal crypts at the base of the tissue (Fig. 1*G*). Interestingly, expression of GREM1 protein was also detected in AKP colonic tumors, with strong staining at the basolateral surface of colonic epithelial cells (Fig. 1*H*). Little or no signal was obtained using isotype control antibodies (Fig. 1, *E* and *F*). These data suggest that low levels of GREM1 protein are detected in normal mouse intestine, with higher levels evident in AKP intestine and tumor tissue.

**Figure 2:**
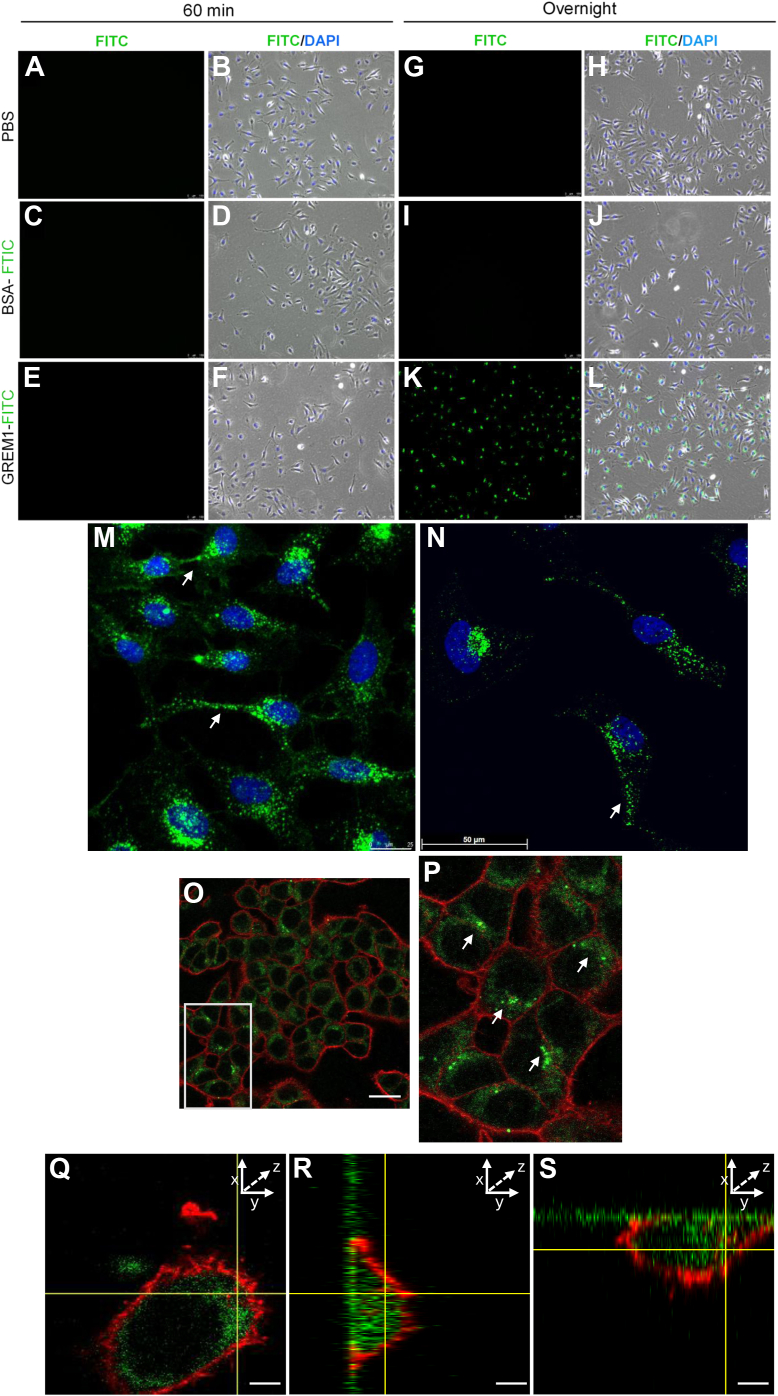
**GREM1 is internalized by HeLa cells.** HeLa cells were treated with PBS, 0.5 μg/ml BSA-FITC or GREM1-FITC in complete medium for 60 min (*A*-*F*) or overnight (*G*-*L*). After incubation, cells were imaged on a DMi8 microscope as described in Methods. Panels show FITC fluorescence (*green*) and overlay with phase contrast and DAPI staining (nucleus, *blue*) (*A*-*L*). Higher magnification images using Leica SP5 Confocal laser scanning microscope are shown in *M*, *N*. GREM1-FITC located in cell extensions is indicated with an arrow. HCT116 cells were incubated with GREM1-FITC (*green*) overnight and then co-stained with phalloidin (*red*) to visualize F-actin filaments (*O*, *P*). Scale bar 50 μm. Z-stack images from *top down* (*x, y axes*, *Q*), side-view (*y, z axes*; *R*), or side-view (*x, z axes*; *S*) with GREM1-FITC (*green*) and phalloidin (*red*) fluorescence evident. Arrows highlight the staining of GREM1-FITC. Scale bars, 5 μm. Data are representative of n = 3 to 5 independent experiments.

Based on these results, we hypothesized that fibroblasts or other cells in the muscularis layer of the intestine express *GREM1* mRNA that is translated to GREM1 protein, which is secreted and taken up by proximal epithelial cells. To interrogate this hypothesis, GREM1-FITC protein was generated and added to HeLa cells. GREM1-FITC was indistinguishable from non-labelled GREM1 in terms of their ability to inhibit BMP2-mediated SMAD1/5/8 phosphorylation (Fig. S1*A*). At 60 min, little or no uptake of GREM1-FITC, or control FITC-BSA was evident (Fig. 2, *A*–*F*). In contrast, overnight incubation led to clear uptake of GREM1-FITC, but not BSA-FITC, into HeLa cells (Fig. 2, *G*–*L*). High-resolution confocal images demonstrated a distinct punctate, perinuclear staining pattern for GREM1-FITC, with staining also detected in cellular extensions and contact points between cells (Fig. 2, *M* and *N*). Confocal imaging clearly showed GREM1-FITC inside the HCT116 cells, beyond the margin of the phalloidin/F-actin staining in the cell cortex (Fig. 2, *O* and *P*). Z-stack images from three different angles confirmed that GREM1 was inside, rather than bound to the surface of HCT116 cells (Fig. 2, *Q*–*S*). Finally, a reduction in surface streptavidin staining was detected using flow cytometry when GREM1-biotin-treated cells were rapidly switched from 4 °C to 37 °C, indicative of GREM1 internalization (Fig. S2, *B* and *C*). Together, these data suggest that GREM1 can bind to the cell membrane and is internalized by cells.

**Figure 3:**
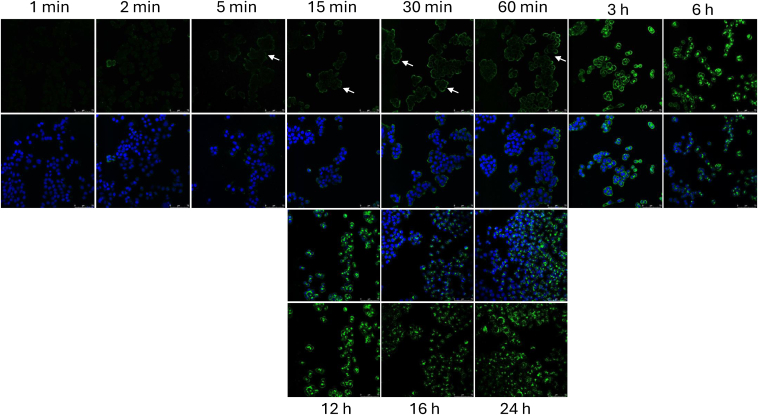
**GREM1 uptake involves accumulation at the plasma membrane.** HCT116 cells were treated with 1 μg/ml GREM1-FITC for the indicated times before washing once with PBS, fixation with 4% PFA and DAPI staining as described in Methods. FITC and DAPI staining were then visualized on a Leica SP5 Confocal laser scanning microscope. Arrows highlight the staining of GREM1-FITC. Scale bar, 25 μm and is shown on *right hand side*.

Binding of GREM1-FITC to HCT116 colorectal cancer epithelial cell membranes became evident between 5 and 15 min, with a ring-like membrane-bound pattern evident at 60 min (Fig. 3). Internalization of GREM1-FITC became evident at 3 to 6 h, with most of the GREM1-FITC detected inside the cells between 16 to 24 h (Fig. 3). Live-cell imaging of GREM1-FITC uptake into HCT116 cells supported these conclusions (Fig. S2 and video). These data suggest that GREM1 internalization is not immediate and may involve GREM1 binding to membrane proteins or receptors prior to internalization.

**Figure 4:**
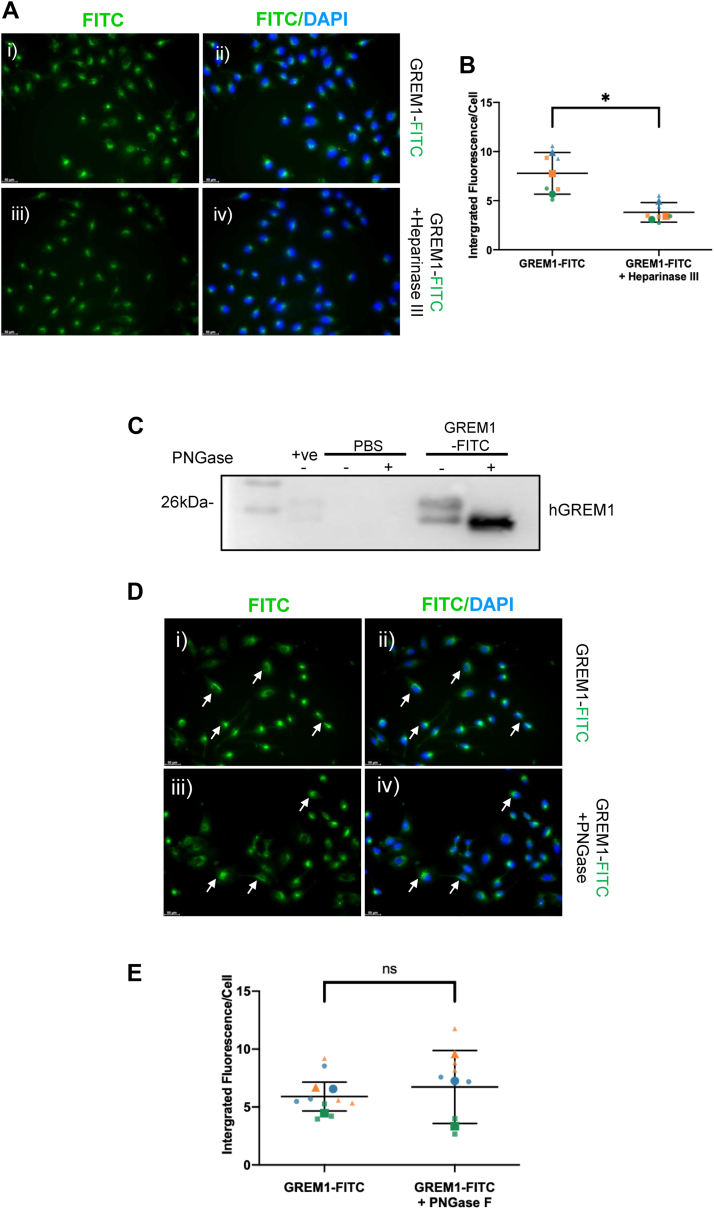
**Heparin sulfate proteoglycans, but not glycosylation, is required for GREM1 uptake into cells.***A*, HeLa cells were preincubated with vehicle or Heparinase III followed by the addition of GREM1-FITC overnight. *C*, deglycosylated GREM1-FITC (500 ng) was added to HeLa cells overnight (*D*, *E*). Arrows highlight the staining of GREM1-FITC. *B – E*, integrated fluorescence per cell was calculated using ImageJ for three images per technical replicate, with n = 3 independent experiments plotted. Large circles, squares, and triangles represent the mean fluorescence intensity from each experiment, and smaller symbols represent the fluorescence intensity of each technical replicate within each individual experiment. Statistical significance was determined using a two-tailed Student’s *t* test (NS, non-significant; ∗, *p* < 0.05).

Several reports have identified that heparin sulphate proteoglycans (HSPGs) are involved in GREM1 signaling and biology (31, 38, 39). Removal of heparin moieties by cell treatment with Heparinase III significantly reduced GREM1-FITC uptake, suggesting that binding or interaction with HSPG-containing membrane proteins is required for GREM1 uptake (Fig. 4, *A* and *B*). GREM1 is also a glycoprotein, with the role of glycosylation suggested to be cell association and binding of GREM1 to the extracellular matrix (40). PNGase deglycosylase treatment of GREM1-FITC led to a downward shift in mobility on SDS-PAGE, confirming deglycosylation (Fig. 4*C*). Deglycosylated GREM1-FITC was taken up in HeLa cells to the same extent as glycosylated GREM1-FITC (Fig. 4, *D* and *E*), suggesting a non-critical role for GREM1 glycosylation for its uptake. These data suggest that while heparin-containing proteins such as HSPGs are required for GREM1 internalization, glycosylation of GREM1 does not appear to be a critical mediator of its binding to and uptake into cells.

**Figure 5:**
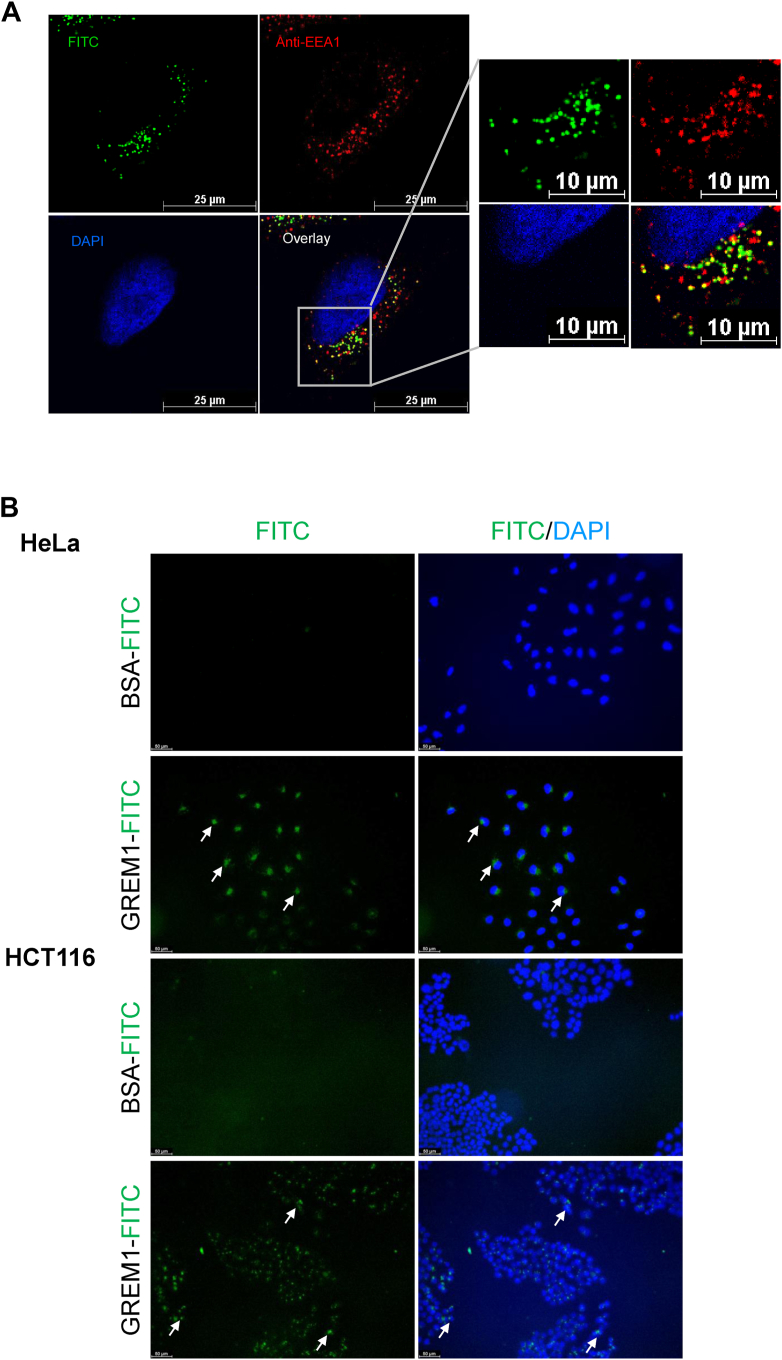
**GREM1localises to the early endosomes andcan be resecreted from cells after endocytosis.***A*, HeLa cells were treated with GREM1-FITC and stained with anti-EEA1 (*red*) and DAPI (*blue*) to visualize the early endosomes and nuclei, respectively. Scale bars represent 25 μm or 10 μm in magnified images. Images are representative of n = 3 independent experiments carried out in duplicate. *B*, conditioned medium (CM) was collected from HeLa cells treated with either FITC-BSA (1 μg/ml) or GREM1-FITC (1 μg/ml) overnight. Fresh HeLa or HCT 116 cells were treated with either CM fraction overnight to measure uptake of resecreted BSA or GREM1-FITC. Arrows highlight the staining of GREM1-FITC. Data are representative of n = 3 independent experiments performed in duplicate. Scale bars, 50 μm.

Confocal microscopy identified a distinct pattern of staining for GREM1-FITC inside cells, with a punctate, perinuclear pattern evident (Figs. 2 and 3), and evidence of GREM1-FITC in filopodia and other cellular extensions (Fig. 2, *M* and *N*). No significant localization of GREM1-FITC to either mitochondria (stained with MitoTracker) or ER (stained with calreticulin) were detected (Fig. S3), with very slight staining evident in the Golgi apparatus using GM130 as a Golgi marker (Fig. S3*C*). The punctate staining pattern was suggestive of lysosomal or endosomal localization. A small amount of GREM1-FITC staining was detected in lysosomes (identified using anti-LAMP1, Sup.Fig. S4). Staining of cells with EEA1, a marker of early endosomes, demonstrated some overlap with GREM1-FITC staining in HeLa cells (Fig. 5*A*). These data suggest that internalized GREM1 partially localizes to the early endosomal compartment. Treatment of HeLa and HCT116 cells with CM captured from GREM1-FITC-treated cells generated green cells (Fig. 5*B*), suggesting that endocytosed GREM1-FITC can be resecreted and taken up into new cells after resecretion. However, no significant overlap in staining of GREM1-FITC was detected when Rab11 was used as a marker of recycling endosomes (Fig. S4*B*), suggesting that resecretion *via* this pathway may not be the primary mechanism for intracellular GREM1 recycling.

**Figure 6:**
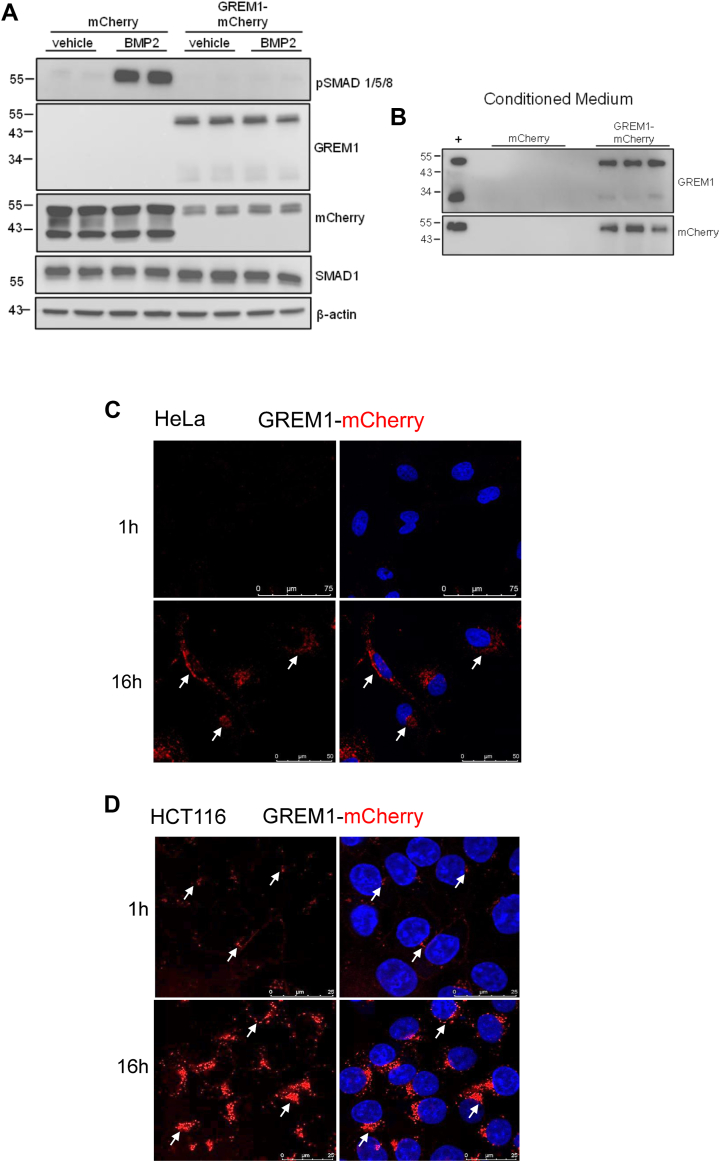
**GREM1-mCherry inhibits BMP2 signaling and is internalized by mammalian cells.***A*. HEK293T cells transfected with either mCherry empty vector or hGREM1-mCherry were treated with vehicle or 5 ng/ml BMP2 at 37 °C for 1 h. *B*. Conditioned medium (CM) was collected from transfected cells and analysed by Western blotting. Cell lysate from transfected HEK293T cells used as positive control (+). Data are representative of n = 3 independent experiments. HeLa (*C*) or HCT116 (*D*) cells were exposed to CM from pCMV-mCherry or pCMV-GREM-mCherry-transfected cells for 1 h or overnight (16 h). Arrows highlight the staining of GREM1-mCherry. Scale bars are indicated in the images. Images are representative of six images per triplicate technical replicate from n = 3 independent experiments.

One potential limitation of our study may be the reliance on commercially available recombinant human GREM1 protein (from Biotechne/R&D Systems). To overcome this, we generated plasmids that express a fusion protein of GREM1 with mCherry (GREM1-mCherry, Fig. 6). GREM1-mCherry was detected at ∼55 kDa in HEK293T cells and inhibited BMP2-stimulated SMAD1/5/8 phosphorylation, confirming functionality (Fig. 6*A*). Similar data were obtained when BMP4 was used to stimulate SMAD1/5/8 phosphorylation (Fig. S1*B*). Serum-free CM collected from transfected HEK293T cells confirmed that GREM1-mCherry was secreted, similar to endogenous GREM1 (Fig. 6*B*). Incubation of HeLa and HCT116 cells with CM from mCherry control or GREM1-mCherry transfected cells demonstrated clear uptake of GREM1-mCherry in overnight-treated cells, with low levels of uptake in HCT116 cells, but not HeLa cells at 60 min (Fig. 6, *C* and *D*). Importantly, the intracellular staining pattern observed with GREM1-mCherry was consistent with that observed with GREM1-FITC, suggesting biological consistency when both commercially sourced rhGREM1 and expressed, secreted, “homemade” GREM1-mCherry were employed (Figs. 2, 3 and 6).

**Figure 7:**
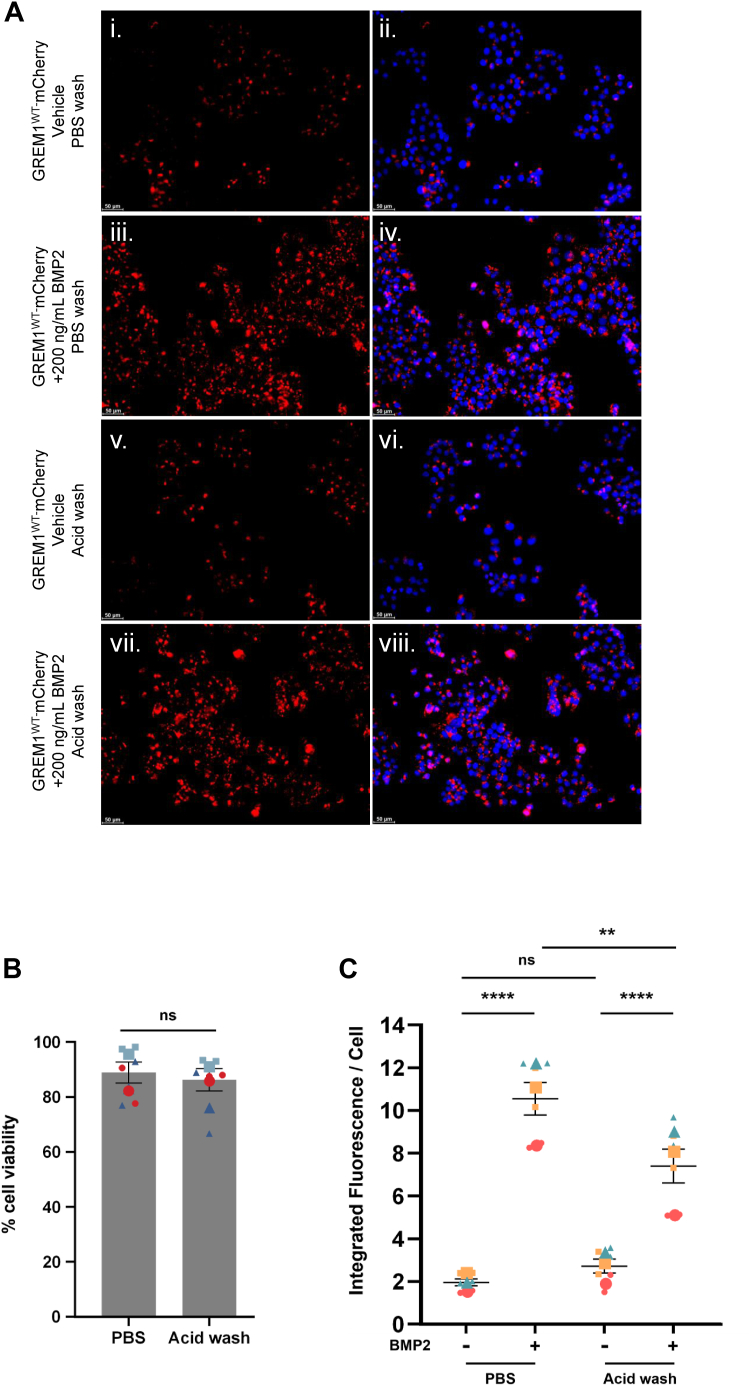
**GREM1 endocytosis is increased by BMP2 and not affected by acid washing.***A*, CM from HEK293T cells transfected with GREM1-mCherry was added to HCT116 cells treated with vehicle (4 mM HCl and 0.1% BSA) or 200 ng/ml BMP2 overnight (16 h). *B*, HCT116 cells were washed with PBS or acid wash buffer. Cell viability was quantified by trypan blue exclusion and expressed as a percentage of total cells. Data are presented as mean ± SEM. Large symbols represent the mean viability from *n* = 3 independent experiments; small symbols represent the average viability of technical replicates. *C*, integrated fluorescence intensity per cell was quantified using ImageJ after blinding the images. Data are presented as mean ± SEM. Large symbols indicate the mean fluorescence intensity from n = 3 independent experiments. Small symbols are representative of the average fluorescence of three images taken per technical replicate. Statistical analysis was carried out using one-way ANOVA. ∗∗, *p* < 0.01; ∗∗∗∗, *p* < 0.001. ns non-significant.

Previous reports have suggested that BMP2 could be transferred between neighboring cells *via* vesicular transport, and that BMP antagonists such as Noggin increased BMP2 transfer between cells (41, 42). Addition of BMP2 to CM-containing GREM1-mCherry increased GREM1-mCherry uptake into HCT116 cells ∼5-fold (Fig. 7, *A* and *C*). To confirm that BMP2 treatment increased GREM1-mCherry internalization *versus* membrane binding, HCT116 cells were treated with an acid wash buffer (glycine pH 3.0) to remove surface-bound proteins (43, 44, 45). Acid wash treatment did not affect overall cell viability compared to cells washed with PBS (Fig. 7*B*). No significant difference in GREM1-mCherry fluorescence intensity was detected in HCT116 cells in the PBS *versus* acid-washed groups (Fig. 7*B* i, ii *versus* v, vi, Fig. 7*C*). Acid wash treatment modestly reduced BMP2-mediated GREM1-mCherry uptake, but the overall increase in GREM1-mCherry internalization was still detected (Fig. 7, *A* and *C*). These data support our conclusion that GREM1-mCherry fluorescence in the absence or presence of BMP2 reflects GREM1 internalization rather than plasma membrane binding.

**Figure 8:**
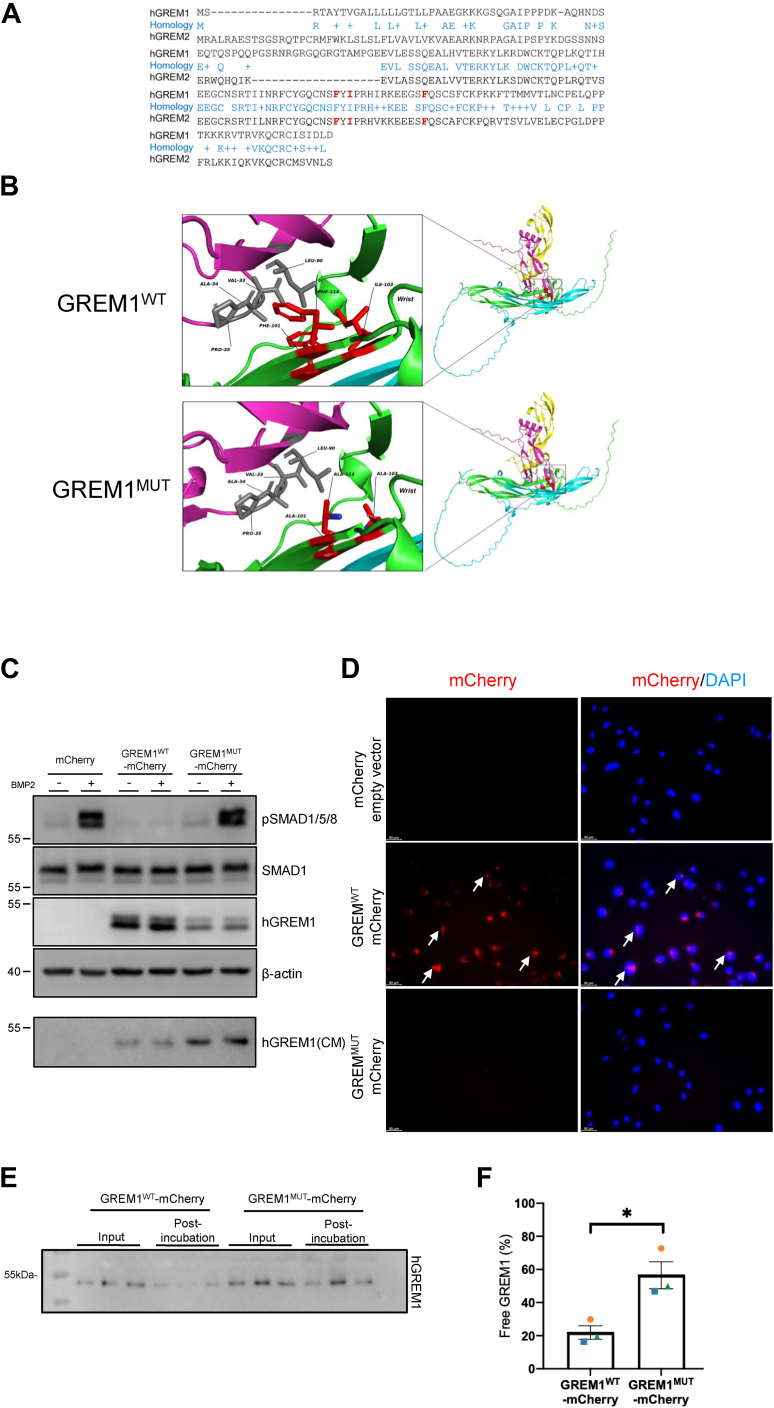
**A mutant GREM1 that cannot bind BMP2 displays reduced internalization.***A*, sequence alignment of human GREM1 and GREM2 (*black text*) with identity and homology indicated (*blue text*). Key GREM1 amino acids identified for BMP binding (F125, I127 and F138) indicated in red. These amino acids cluster in the central two β-strands, with F125 and I127 at the end of strand two and F138A close to the start of strand 3. All three of these amino acids were mutated to Alanine in the GREM1 mutant. *B*, alpha fold three predicted structures of GREM1^WT^ and GREM1^Mut^ bound to BMP2 with the positions of the three amino acids F125 (Phe101), I127 (Ile103) and F138 (Phe114) in the β-strands indicated. The numbering of the amino acids differs in the AlphaFold three structure as the mature GREM1 protein lacking the secretion signal (amino acids 1-24) was used to generate the predicted structure. *C*, HEK293T cells were transfected with plasmids expressing mCherry, GREM1^WT^-mCherry and GREM1^MUT^-mCherry followed by treatment with vehicle (4 mM HCl and 0.1% BSA) or 5 ng/ml BMP2 for 1 h before adding 1 ml serum-free media for 4 h to harvest conditioned medium (CM). *D*, CM collected from HEK293T cells transfected with plasmids expressing mCherry, GREM1^WT^-mCherry or GREM1^MUT^-mCherry was added to HeLa cells overnight. (n = 3 independent experiments). Arrows highlight the staining of GREM1-FITC. *E*, CM was collected and analyzed by SDS-PAGE. GREM1^WT^-mCherry or GREM1^MUT^-mCherry were detected by anti-hGREM1 antibody before addition of CM to HEK293 cells (Input) or after overnight incubation (post-incubation). Images are representative of n = 3 independent experiments carried out in triplicate. *F*, Image J was used to calculated GREM1 band intensities, and the ratio of GREM1 in post-incubation/input samples was calculated to estimate the percentage of free GREM1 remaining in the CM. Symbols represent means of triplicate values from n = 3 independent experiments with error bars indicating SEM. Statistical significance was calculated using a two-tailed Student’s *t* test ∗, *p* < 0.05.

Previous research by Nolan *et al.* identified that three specific amino acids (F125/I127/F138) were required for robust binding of GREM2 (another related BMP antagonist of the DAN family with 60% sequence identity to GREM1) to BMP/GDF targets (46). Sequence alignment of GREM1 and GREM2 demonstrated that these amino acids are conserved in GREM1 and cluster together in β-sheet strands 2 and 3 (Fig. 8*A*). These three amino acids were mutated to Ala to generate a F125A/I127A/F138A GREM1 mutant (GREM1^Mut^) that were expressed, along with wild-type GREM1, as mCherry fusion proteins (GREM1^WT^-mCherry, GREM1^Mut^-mCherry, Fig. 8). A model of GREM1 binding to BMP2 generated using AlphaFold3 displays the effect of these three amino acid mutations on the predicted binding surfaces between GREM1 and BMP2, suggesting a disruption of stabilizing hydrophobic interactions (Fig. 8*B*). Both GREM1^WT^-mCherry and GREM1^Mut^-mCherry were expressed in HEK293 cells, with lower levels of GREM1^Mut^-mCherry detected in cell lysates, but higher levels detected in the CM, suggesting more efficient secretion of this GREM1 mutant (Fig. 8*C*). Addition of BMP2 demonstrated a clear increase in SMAD1/5/8 phosphorylation in control cells that was completely inhibited in GREM1WT-mCherry-transfected cells (Fig. 8*C*). In contrast, cells transfected with GREM1^Mut^-mCherry demonstrated little or no inhibition of BMP2-induced Smad1/5/8 phosphorylation (Fig. 8*C*). A similar reduction in the ability of CM containing GREM1^WT^ v GREM1^Mut^-mCherry to inhibit BMP2 was observed when C2C12 cells transfected with a BMP response element (BRE) luciferase reporter (47) were utilized (Fig. S1*C*).

**Figure 9:**
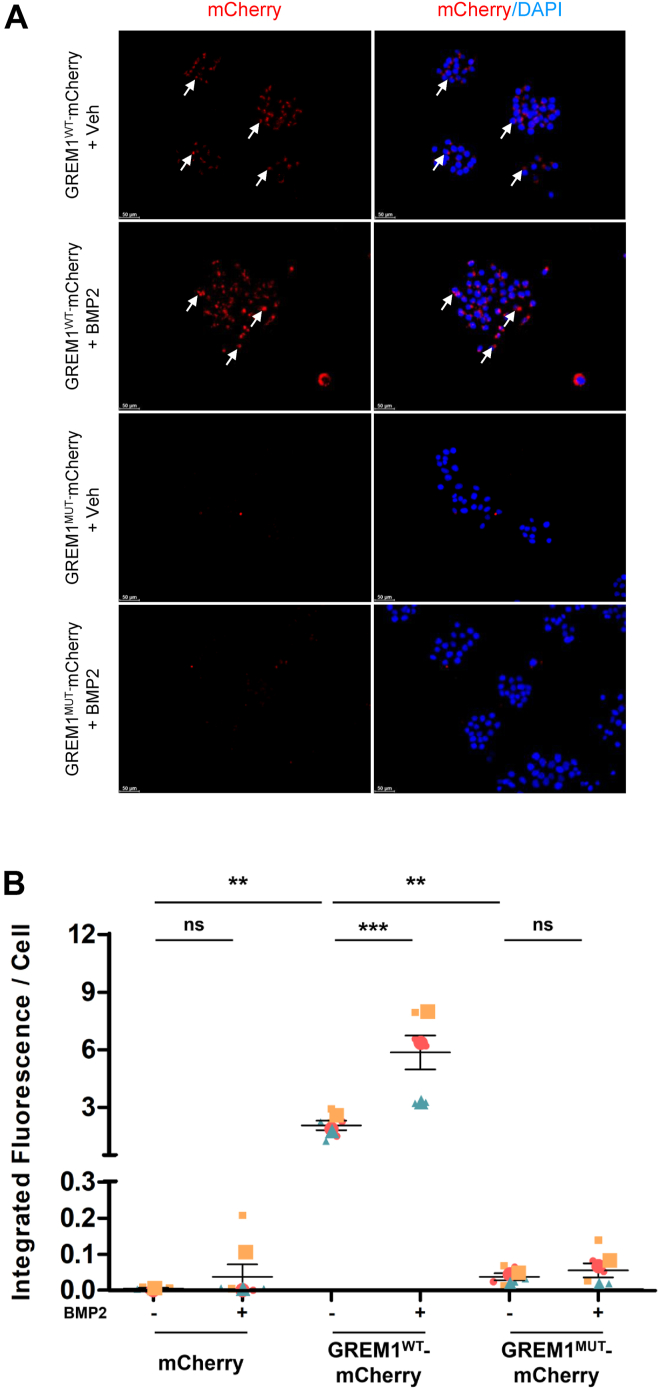
**BMP2 increases GREM1^WT^ but not GREM1^Mut^-mCherry uptake into HCT116 cells.***A*, conditioned medium from HEK293 cells transfected with GREM1^WT^-mCherry or GREM1^Mut^-mCherry added to HCT116 cells in the presence of either vehicle (4 mM HCl and 0.1% BSA) or 200 ng/ml BMP2 overnight for 16 h. Arrows highlight the staining of GREM1-FITC. Scale bars represent 50 μm. *B*, integrated fluorescence intensity per cell was quantified and plotted by ImageJ and GraphPad Prism after blinding the images. Data are presented as mean ± SEM. Large symbols indicate the mean fluorescence intensity from n = 3 independent experiments. Small symbols represent the average of duplicate wells, with three images taken per well. Statistical analysis was determined using one-way ANOVA followed by Bonferroni *post hoc* test. (∗∗, *p* < 0.01; ∗∗∗, *p* < 0.001).

These data suggest that GREM1^Mut^-mCherry is resistant to BMP2 binding and may therefore be used as a tool to determine the requirement of BMP binding for GREM1 action. The uptake of GREM1^WT^-mCherry *versus* GREM1^Mut^-mCherry into cells was then compared. The addition of CM containing GREM1^WT^-mCherry to HeLa cells demonstrated clear uptake after overnight incubation (Fig. 8*D*). Surprisingly, uptake of GREM1^Mut^-mCherry was much lower compared to GREM1^WT^-mCherry (Fig. 8*D*). The lower intensity of mCherry fluorescence seen with GREM1^Mut^-mCherry was not caused by protein degradation, as GREM1^Mut^-mCherry protein was detected in the CM after removal from cells post-incubation at a similar level to the pre-incubation CM (Fig. 8, *E* and *F*). In contrast, lower levels of GREM1^WT^-mCherry were detected in post-*versus* pre-incubation CM, likely due to partial uptake into cells (Fig. 8, *E* and *F*). Addition of BMP2 significantly increased GREM1^WT^-mCherry uptake into HCT116 cells but had no effect on GREM1^Mut^-mCherry uptake, consistent with an inability of this mutant form of GREM1 to bind to BMP2 (Fig. 9). These data support the conclusion that BMP binding is required for GREM1 uptake into mammalian cells.

## Discussion

Our report identifies a novel mechanism of GREM1 uptake into intestinal epithelial cells, a process that is both enhanced by, and requires BMP binding. Canonical GREM1 signaling involves binding to BMP agonists in the extracellular matrix leading to inhibition of BMP-mediated signaling. Our report extends this model, suggesting that internalization of GREM1-BMP complexes may also contribute to attenuation of extracellular BMP signaling by GREM1.

Differential GREM1 mRNA and protein were detected in stromal fibroblasts/muscularis layer *versus* epithelial cells at the base of the crypts (Fig. 1). One explanation for this phenomenon is that the transcriptional landscape (including epigenetic marks of permissive chromatin such as H3K27Ac) is more favorable to GREM1 transcription in fibroblasts compared to epithelial cells. A second possibility is that transcription factors required to bind to cis-regulatory elements on the GREM1 promoter are abundant in fibroblasts but not present in epithelial cells. A third scenario is that transcriptional repressors may be present in epithelial cells that limit GREM1 expression compared to adjacent fibroblasts. The model of GREM1 protein expression and secretion from stromal fibroblasts and uptake into adjacent epithelial cells adds to our understanding of BMP/GREM1/Wnt morphogen gradient signaling in the intestinal stem cell niche (25). Overexpression of GREM1 in mouse intestinal epithelial cells disrupts the BMP morphogen gradient and leads to expansion of CD55^+^ Wnt2b^+^ fibroblasts, leading to remodeling of the stromal cell compartment and ectopic crypt formation (25). Thus, exquisite control of GREM1 expression is required to ensure balanced BMP/Wnt morphogen gradients and normal intestinal homeostasis.

Our data demonstrate that GREM1 protein expression is upregulated in the AKP transgenic mouse model of colorectal cancer, which expresses APC/KRAS/p53 mutations commonly found in human colorectal cancer (35, 36, 37). GREM1 protein was detected in the muscularis layer and epithelial cells of the colonic crypts, as well as in tumors that formed in these mice due to AKP transgene expression (Fig. 1). Our data suggest that upregulation of GREM1 protein is also a feature of tumors formed by these genetic alterations in mice. To confirm the relevance of these data for human CRC, screening tissue microarrays from human colorectal cancers for GREM1 protein expression and comparing them to RNA levels using spatial transcriptomics would be the logical next step. However, currently available GREM1 antibodies show poor sensitivity and specificity for human GREM1, and existing data in the literature reporting GREM1 protein expression in human tissues should be interpreted with caution.

GREM1 uptake into cells appeared to take hours rather than minutes, with the majority of FITC-GREM1 outside the cell bound to the plasma membrane at the 3 h timepoint (Figs. 2 and 3). The dynamics of GREM1 uptake is somewhat slow in comparison to ligand-bound GPCR internalization which occurs within 20 to 30 min (48). Glycosylation of GREM1 was not required for internalization but removal of cellular HSPGs reduced GREM1 uptake by approximately 50% (Fig. 4). Reports in the literature have suggested that GREM1 can bind to receptor tyrosine kinases (RTKs) such as VEGFR2 (30), EGFR (34)) and FGFR1 (33), triggering downstream signal transduction. Using a BRET-based approach monitoring activation of G-protein isoforms, GREM1 failed to activate any G-protein in either HEK293 or U251 glioblastoma cells, suggesting that GREM1 is not binding to a GPCR at the plasma membrane (data not shown). The reduction in GREM1 internalization after Heparinase III treatment of cells suggests that some heparin sulfate-containing protein is involved in GREM1 binding at the plasma membrane (Fig. 4). HSPG-bound BMPs are likely a localized reservoir for extracellular BMP, potentially serving to increase co-localization of GREM1 and BMP in the extracellular space, which may facilitate enhanced GREM1-BMP binding and subsequent internalization (49, 50, 51, 52). This may provide an explanation for the decrease in GREM1 uptake after heparinase treatment (Fig. 4). While RTKs such as FGFR1 signaling also requires HSPGs (53), we cannot exclude the possibility that other membrane HSPGs such as syndecans or glycosylphosphatidylinositol-anchored proteoglycans (glypicans) may be candidate receptors for GREM1 (54).

Endocytosed GREM1 does not localize exclusively to a specific subcellular compartment such as the Golgi or endoplasmic reticulum but seems to be distributed partially across the early endosomal and lysosomal compartments (Figs. 2, 5*A* and S4). GREM1 can be resecreted once taken up into mammalian cells (Fig. 5*B*) suggesting a dynamic model where GREM1 can be endocytosed and then resecreted for potential uptake into neighboring epithelial cells. These data raise the question: what is the biological significance of GREM1 secretion and internalization by cells? Given the somewhat slow rate of GREM1 endocytosis and the requirement for HSPGs for this process (Fig. 4*A*), the possibility exists that GREM1 cellular internalization may occur as part of a complex with membrane receptors such as RTKs or other HSPG-containing proteins. Future experiments will be needed to identify whether binding to this putative receptor represents a previously unappreciated “non-canonical” signaling modality for GREM1, independent of BMP antagonism.

GREM1-BMP2 complexes were more efficiently endocytosed by HCT116 cells (Fig. 7). These data are supported by previous reports from the Wolfl group, who demonstrated that BMP2 uptake into HeLa cells was increased by Noggin, but decreased by Chordin, both BMP antagonists (41). Based on the enhancement of GREM1 uptake by BMP2, a transcytosis model of morphogen signaling model may exist here, similar to that described for other BMP antagonists such as Noggin (41). However, it is not clear whether complexes of GREM1 and BMP2, once endocytosed, can be further dissociated inside the cell, thus allowing free GREM1 and BMP2 resecretion. Indeed, we propose the idea that the endocytosis of GREM1 as part of a complex with BMP targets may reduce the local concentration of BMP in the extracellular matrix, enhancing GREM1 antagonism. This model is consistent with local gradients of GREM1 regulating extracellular BMP signaling, as well as Wnt morphgen signaling in the intestinal stem cell niche (25). Data from the acid wash experiments, which should strip membrane-bound GREM1 and GREM1/BMP2 complexes suggest that fluorescence signals detected represent GREM1 internalization and not external membrane-associated GREM1 (Fig. 7).

The absolute requirement for BMP2 binding to GREM1 for cellular uptake was demonstrated by the dramatic reduction in the uptake of the non-BMP2 binding GREM1^Mut^ protein even in the absence of exogenous BMP2 (Figs. 8 and 9). This attenuation of uptake was not due to lower stability of the GREM1^Mut^ protein, as equal levels of GREM1^Mut^ were detected in the CM pre- and post-incubation (Fig. 8, *E* and *F*). Several groups have identified receptor tyrosine kinases such as VEGFR2, EGFR and FGFR1 as targets for GREM1 (30, 33, 34). To date, it has not been clear whether the ability of GREM1 to bind to BMP ligands modulates this RTK signaling. The GREM1^Mut^, which we clearly demonstrated, does not bind to BMP2 in both HCT116 (Fig. 8*C*) and C2C12 cells (Fig. S1*C*) may be a valuable reagent to further refine our understanding of GREM1-RTK signaling in cancer and other diseases.

The increase in GREM1 endocytosis in the presence of BMP2 would perhaps argue against the requirement for GREM1 binding to a specific membrane receptor prior to internalization, as BMP2 binding to GREM1 would, in theory, reduce GREM1 binding to this putative receptor. The structure of the GREM1-BMP2 complex is predicted to be a collection of ‘fibril-like’ open-ended oligomers with interacting ⍺-helixes of BMP2 and GREM1 (46, 55). One possibility could be that these large “daisy-chain”-like complexes more readily bind non-specifically to the plasma membrane, leading to enhanced endocytosis. However, we cannot exclude the possibility that an as-yet-unidentified heparin-containing membrane receptor may be involved in enhanced GREM1-BMP2 uptake. Interestingly, when these experiments were repeated in serum-free medium, ∼ 2-fold higher GREM1 uptake was detected (Fig. S5). A reduction in GREM1 endocytosis in the presence of BMP2, with more marked GREM1 binding to cell membranes and cell-cell junctions was also detected (Fig. S5). These data suggest that protein (or other) components in FBS may be competing with GREM1, thereby reducing cell binding. Conversely, GREM1-BMP2 complexes appear to bind to but not enter cells in the absence of FBS, suggesting that unknown ingredients of FBS are required for full uptake of GREM1-BMP2 complexes.

In conclusion, we identify a novel mechanism of GREM1 protein secretion and uptake into cells that require BMP2 binding. Future experiments will determine the significance of this GREM1-BMP2 uptake mechanism in other cell-types, tissues, and human diseases.

## Experimental procedures

### Cell culture

HEK293T cells were cultured in Dulbecco's Modified Eagle Medium (DMEM) containing pyruvate, 1 g/L glucose (Gibco, Cat. No: 31885023), supplemented with 10% FBS (Gibco, UK,

Cat. No: 10082147) and 1 mM HEPES (SIGMA-ALDRICH, Cat. No: H0887). HEK293Tcells were maintained in a T75-cm^2^ flask at 37 °C, 5% CO_2_ and plated on 6-well plates or 10-cm^2^ dishes for treatments. HCT116 human colon cancer cells were cultured in McCoy’s 5a.

Medium Modified with L-Glutamine (Gibco, Cat. No: 26600023), supplemented with

10% FBS (Gibco, Cat. No: 10082147) and 1 mM sodium pyruvate (Gibco, Cat. No: 11360070). HCT116 cells were maintained in T75 cm^2^ flask at 37 °C, 5% CO_2_. HeLa cells were cultured in DMEM containing low glucose, pyruvate, and L-glutamine (Gibco, Cat. No: 31885023), supplemented with 1× non-essential amino acids (Gibco, Cat. No: 11140035) and 10% FBS (Gibco, Cat. No: 10082147). HeLa cells were grown in a T75-cm^2^ flask at 37 °C, 5% CO_2_. C2C12-BRE (BMP response element) cells were a generous gift from Professor Gareth Inman (Beatson Institute for Cancer Research, University of Glasgow). Cells were maintained in DMEM (Gibco, Cat. No: 31885023) supplemented with 10% FBS (Gibco, Cat. No: 10082147) in T175 cm^2^ flasks at 37 °C in a humidified 5% CO_2_ incubator. All cell lines were screened every month and confirmed as negative for *Mycoplasma* using a commercially available PCR test kit.

### HEK293 transfection

HEK293T cells were transfected when they reached 60% confluence in 10 cm^2^ dishes well plates. Each dish was transfected with 600 μl of Opti-MEM (Gibco, Cat. No: 31985047) and 18 μl Lipofectamine 2000 Invitrogen, Cat. No: 11668019) transfection reagent per dish. The appropriate volume of Lipofectamine 2000 and Opti-MEM MasterMix were mixed followed by 5 min incubation at RT. Then 6 μg of plasmid per dish were added along with 600 μl of Opti-MEM in MasterMix followed by 20 min incubation at RT. An additional 12 ml of DMEM supplemented with 10% FBS was added to the final mixture media before adding to cells. Medium was changed after 5 h incubation at 37 °C and cells were harvested after 24 h or 48 h post-transfection.

### Protein extraction and Western blotting

Total protein was extracted using RIPA buffer (50 mM Tris-HCl, pH 7.4, 0.5% (v/v) sodium deoxycholate, 150 mM sodium chloride (NaCl, Cat. No: S23020), 1% (v/v) Triton X-100 Surfact-Amps Detergent Solution (Triton X ThermoFisher Scientific, Cat. No: 85111) and 1 mM EDTA (SIGMA-ALDRICH, Cat. No: E5134). Prior to adding to cells, RIPA buffer was supplemented with 250 μM sodium orthovanadate (Na3VO4, SIGMA-ALDRICH, Cat. No: 450243), 40 mM β-glycerolphosphate (SIGMA-ALDRICH, Cat. No: 50020), 1 mM sodium fluoride (NaF, SIGMA-ALDRICH, Cat. No: 450022), 2 μM microsystin-LR (Enzo Life Sciences, Cat. No: ALX-350-012), 1 mM phenylmethanesulfonylfluoride (PMSF, SIGMA-ALDRICH, Cat. No: 329-98-6) and 1 x protease inhibitor cocktail (SIGMA-ALDRICH, Cat. No: P8340). Cell lysates were diluted with 2 × Laemmli buffer containing β-Mercaptoethanol before boiling at 95 °C for 5 min. Cell lysates were loaded into 10% or 15% (v/v) SDS-PAGE gels. Proteins were transferred to PVDF membranes, which were blocked with 3% BSA/TBST for 1 h. Membranes were incubated in the various primary antibodies overnight at 4 °C (Anti-human Gremlin1 (R&D systems, Cat. No: AF956), Anti-mCherry antibody (Abcam, Cat. No: ab125096), Anti-β-actin (Cell Signalling, Cat. No: 3700S), Anti-PhosphoSMAD1(Ser463/465)/SMAD5 (Ser463/465)/SMAD8 (Ser465/467) (Cell Signalling, Cat. No: 13820S), Anti-SMAD1 (Cell Signalling, Cat. No: 6944S). Membranes were then washed 3 times with 1X TBST and incubated with the respective secondary antibody (1:10,000) for 1 h at RT. After three washes in 1 X TBST, membranes were exposed to enhanced chemiluminescence (ECL) reagents (Merck, Cat. No: WBKLS0050), and bands were visualized using the Genesys (G: BOX) imaging system.

### Immunofluorescence

Cells were seeded on either plastic plates or glass coverslips. Experimental treatments were initiated when cells were at 30 %-40% confluency. Once fixed using 4% (w/v) PFA (SIGMA-ALDRICH, Cat. No: 158127) or 10% (w/v) formaldehyde (ThermoFisher Scientific, Cat. No: 28908), cells were permeabilized with 0.1% Triton-X in PBS and blocked with PBS contain 0.5% (v/v) Triton-X and 2% (w/v) BSA for 2 h at 4 °C. Cells were incubated at 4 °C overnight in primary antibody (Anti-EEA1 1:100, Cell Signaling, Cat. No: 2411S), MitoTracker Red (Invitrogen, Cat. No: M7512), Anti-Calreticulin (Abcam, Cat No: ab92516), Anti-GM130 (1:500, BD Transduction Laboratories, Cat. No: 610822), Anti-LAMP1 (1:50, Abcam Cat. No: ab25630), Anti-Rab 11a (1:100, ThermoFisher Scientific, Cat. No: 700184)made up in blocking buffer. After three × PBS washes, Alex-568 labelled secondary (rabbit anti-mouse) antibody was added at a 1:1000 dilution in blocking buffer, for 1 h at RT. Cells were washed three ×times with PBS, and coverslips were then placed onto glass slides using VECTASHIELD Mounting Medium with DAPI (Cat. No: H1200) and left to dry overnight at RT protected from light until imaging. Images were captured under a Leica DMi8 microscope or SP5.

### rhGREM1 biotinylation and internalization assay

Fluorescently labeled, biotinylated rhGREM1 (0.5 μg/ml) was added to 1 × 10^4^ HCT16 cells and incubated at 37 °C for 1.5 h or 16 h. Cells were fixed in 4% (w/v) PFA, washed with 1 × PBS and the cell surface was stained with phalloidin eFluor 450 (Invitrogen). Z-stacks were acquired by confocal microscopy using a Leica STELLARIS TCS microscope. Fluorescence signals were detected *via* HyD GaAsP spectral detectors between 420-460 nm and 620-680 nm as appropriate. An HC PL APO 100×/1, 40 Oil STED objective was used for image acquisition. Fluorescence images were collected at a 16-bit depth 1024 × 1024-pixel resolution format and a scanner speed of 600 Hz. LAS X 4.7.0.28176 was used for image acquisition and processing. Antigen internalization was quantified using ImageJ and IMARIS software.

### Flow cytometry

HCT116 cells (1 × 10^6^) were incubated with fluorescently labeled, biotinylated rhGREM1 at 0.5 μg/ml and a LIVE/DEAD marker (Fixable Viability Dye eFluor 780, eBioscience) for 15 min on ice. Cell suspensions were washed with cold 1x PBS, split into two separate samples and resuspended in either 37 °C or ice-cold HBSS, and incubated at respective temperatures for 16 h overnight. Cells were fixed in 4% (w/v) PFA, and the remaining surface GREM1 was counterstained with Streptavidin eFluor 450 (Invitrogen). Samples were analyzed on a BD FACSCanto II.

### Cell viability assays

HCT116 cells were cultured for 48 h at 37 °C in complete medium. Cells were then subjected either to three washes with ice-cold PBS (30 s each, control) or to two washes with acid buffer (0.2 M glycine, 0.15 M NaCl, pH 3.0; 30 s each) followed by a single wash with ice-cold PBS (45). Cell viability was determined by trypan blue exclusion using a hemocytometer and expressed as a percentage of total cells.

### Immunohistochemistry

FFPE sections (5 μm) were deparaffinized in xylene and rehydrated through a graded ethanol series. Endogenous peroxidase activity was quenched by incubating the sections in 3% (v/v) hydrogen peroxide in PBS for 20 min. Antigen retrieval was conducted by heating the sections in 10 mM sodium citrate buffer (pH 6.0), followed by cooling at RT. Tissue sections were then permeabilized and blocked with 1% (v/v) normal rabbit serum (Vectastain Elite ABC kit, Vector Laboratories, Cat. No: PK6105) in 2.5% BSA – 0.3% Triton-X PBS. To further minimize non-specific binding, an Avidin/Biotin Blocking Kit (Vector Laboratories, Cat. No: SP2001) was applied as per the manufacturer’s protocol. Sections were then incubated overnight at 4 °C with a primary anti-mouse GREM1 (1:200, R&D Systems, Cat. No: AF956) or an isotype control (goat normal IgG, 1:200, Santa Cruz, Cat. No: AB108C) in a humidified chamber. Following washing with 0.1% PBS Tween, a secondary biotinylated rabbit anti-goat antibody (1:250, Vectastain Elite ABC kit, Vector Laboratories, Cat. No: PK6105) was added for 1 h at RT, followed by incubation with Streptavidin peroxidase (1:250, Vector Laboratories, Cat. No: SA5004) for 30 min. Visualization was performed using 3,3′-diaminobenzidine (DAB) substrate (DAB substrate kit, Abcam, Cat. No: ab64238), and the reaction was terminated in deionized water. Sections were counterstained with hematoxylin for 15 s, dehydrated through graded ethanol solutions, cleared in xylene, and mounted with DPX. Sections were scanned using Leica AT2 Aperio scanner and images captured using Leica Aperio ImageScope.

### GREM1-FITC generation and treatment

Fluorescein isothiocyanate (FITC)-labeled recombinant human (rh) GREM1 (R&D Systems, Cat. No: 555190-GR-050) was generated using a FITC Conjugation Kit (Abcam, Cat. No: ab102884). This resulted in a 183 μg/ml solution of GREM1-FITC. GREM-FITC was directly diluted into the cell culture medium with different concentrations used in specific experiments (0.5 μg/ml, 1 μg/ml). Cells were protected from light prior to imaging, and GREM1-FITC conjugates were stored at 4 °C.

### Conditioned medium treatments

After 24 h or 48 h of HEK293T cell transfection, conditioned medium (CM) was collected by washing the cells once with PBS, followed by the addition of serum-free medium for 5 h at 37 °C. The CM was centrifuged at 1200 rpm (13,684*g*) for 10 min to remove any floating cells or cellular debris. The CM was then either applied to fresh cells or snap-frozen in liquid nitrogen and stored at −80 °C for later use. Ten % FBS (Gibco, UK, Cat. No: 10082147) and recombinant human (rh) BMP2 (R&D system, Cat. No: 355-BM-050/CF) were employed to measure the effect on GREM1 internalization. The experiments were conducted in both HCT116 and HeLa cells, and GREM1 was expressed as a secreted GREM1^WT^ or GREM1^Mut^-mCherry fusion protein. CM was supplemented with or without 10% FBS and then incubated with vehicle (4 mM HCl and 0.1% BSA) or 200 ng/ml BMP2 overnight (16 h). Following incubation, cells were washed with PBS, fixed and stained with DAPI (Thermo Fisher Scentific, Cat. No: D1306) at a concentration of 1 μg/ml in PBS for 20 min. Imaging was performed using a DMi8 microscope at 20 × magnification.

### Fluorescence quantification of GREM1-FITC and GREM1-mCherry

GREM1-FITC or GREM1-mCherry integrated cell fluorescence was measured using ImageJ. Images displaying only FITC or mCherry fluorescence were used as quantification controls. Before measurement, all images were blinded, and the integrated intensity per cell was calculated with background subtraction. The sum of the integrated density within the selected regions was measured by ImageJ. The total number of cells in each image was counted, with partial cells at the image boundaries counted only on the top and left sides. The integrated fluorescence intensity per cell was then determined by dividing the total integrated density by the number of cells. After completing the quantification, the data were unblinded and subsequently analyzed and plotted using GraphPad Prism (Version 9.1.0).

### PNGase treatment of GREM1

The removal of high mannose, hybrid, and complex oligosaccharides from GREM1-FITC was achieved using the PNGase F enzyme kit (Biolabs, Cat. No: P0704S). One μg of rhGREM1 or GREM1-FITC was combined with 2 μl of GlycoBuffer 2 (10×),100 U of PNGase F and H_2_O to make a 20 μl volume. The reaction mixture was gently mixed and incubated at 37 °C for 2 h. After stopping the reaction with 2 × Laemmli buffer and boiling at 95 °C, deglycosylation of GREM1-FITC was verified accelerated GREM1 protein migration on Western blot (WB). For cell treatments, a parallel reaction without PNGase F, referred to as control GREM1-FITC, was run. Deglycosylated GREM1-FITC internalization experiments were conducted using HeLa cells that were seeded in a 12-well plate and grown to 30 to 40% confluency. Cells were treated overnight with 1 μg/ml of either control or deglycosylated GREM1-FITC. Subsequently, cells were fixed, stained with DAPI, and imaged using a DMi8 microscope.

### Statistical analysis

Graphs were generated and statistical analysis was conducted using GraphPad Prism 5.0 software. Data were plotted according to the format to highlight interexperimental reproducibility and variability (56). The statistical significance of two-group comparisons was assessed using Student's *t* test, while comparisons involving three or more groups were analyzed using One-way ANOVA followed by Bonferroni *post hoc* test. Each experimental group comprised a minimum of three samples, and experiments were repeated at least n = 3 times unless otherwise specified. *p* values were calculated and present as ∗ *p* < 0.05; ∗∗*p* < 0.01; ∗∗∗*p* < 0.001; ∗∗∗∗*p* < 0.0001.

## Disclosure statement

Apart from Figure 9*B*, no generational AI software was used to generate figures or text for this manuscript.

## Data availability

All data in this manuscript are available upon request to the corresponding author (d.brazil@qub.ac.uk).

## Supporting information

This article contains supporting information.

## Conflict of Interest

The authors declare that they do not have any conflicts of interest with the content of this article.
