# Supplementary material for: Identification of a novel GREMLIN1 uptake pathway in epithelial cells that requires BMP binding
Source: J Biol Chem. 2025 Sep 29;301(11):110780. doi: 10.1016/j.jbc.2025.110780 (PMC12597263; doi:10.1016/j.jbc.2025.110780)
Supplement: Supporting Video File [file mmc1.pptx]

## Slide 1
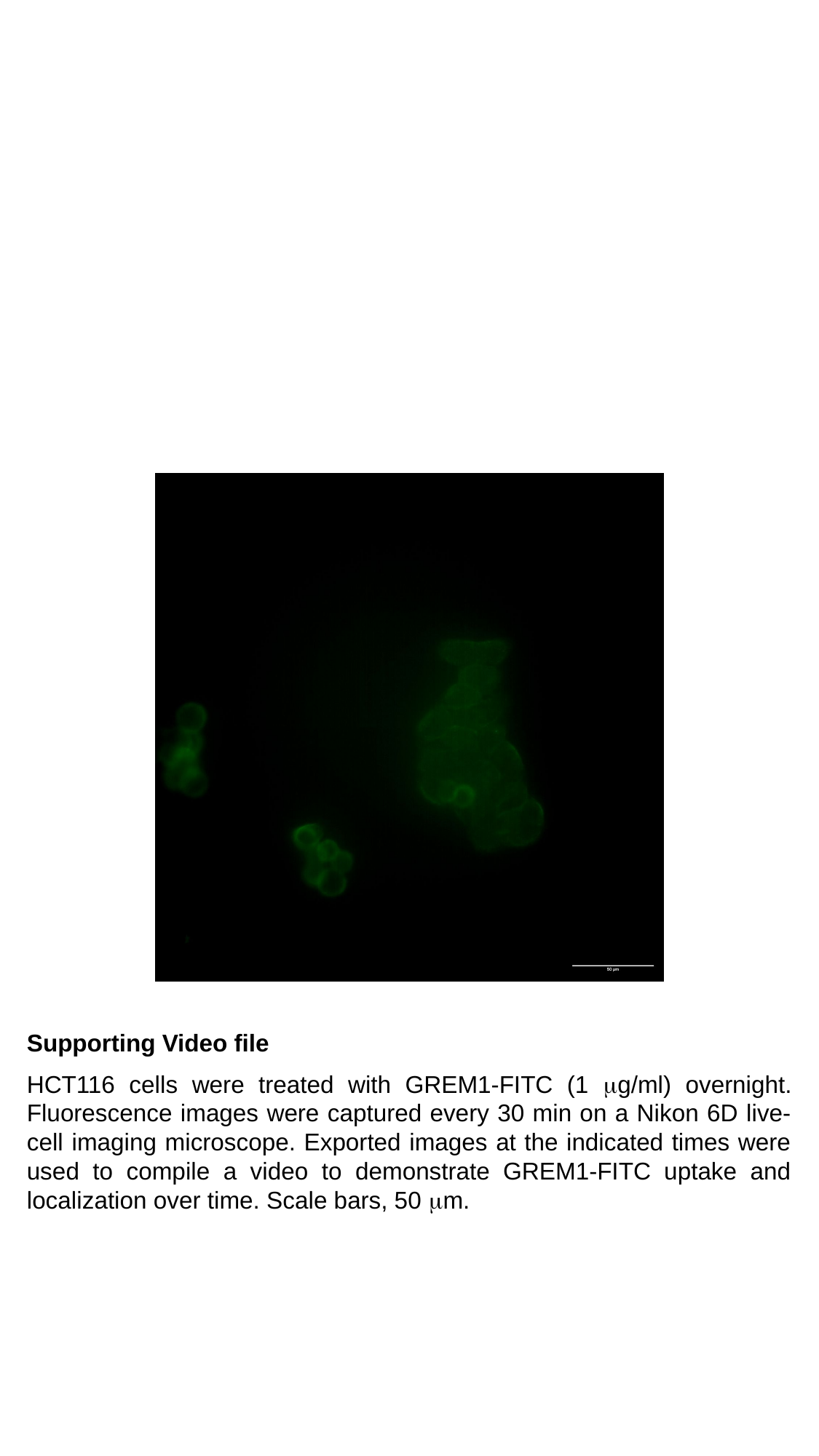

Supporting Video file
HCT116 cells were treated with GREM1-FITC (1 mg/ml) overnight. Fluorescence images were captured every 30 min on a Nikon 6D live-cell imaging microscope. Exported images at the indicated times were used to compile a video to demonstrate GREM1-FITC uptake and localization over time. Scale bars, 50 mm.
