# Supplementary material for: Identification of a novel GREMLIN1 uptake pathway in epithelial cells that requires BMP binding
Source: J Biol Chem. 2025 Sep 29;301(11):110780. doi: 10.1016/j.jbc.2025.110780 (PMC12597263; doi:10.1016/j.jbc.2025.110780)
Supplement: Supporting Figure S1 [file mmc2.pdf]

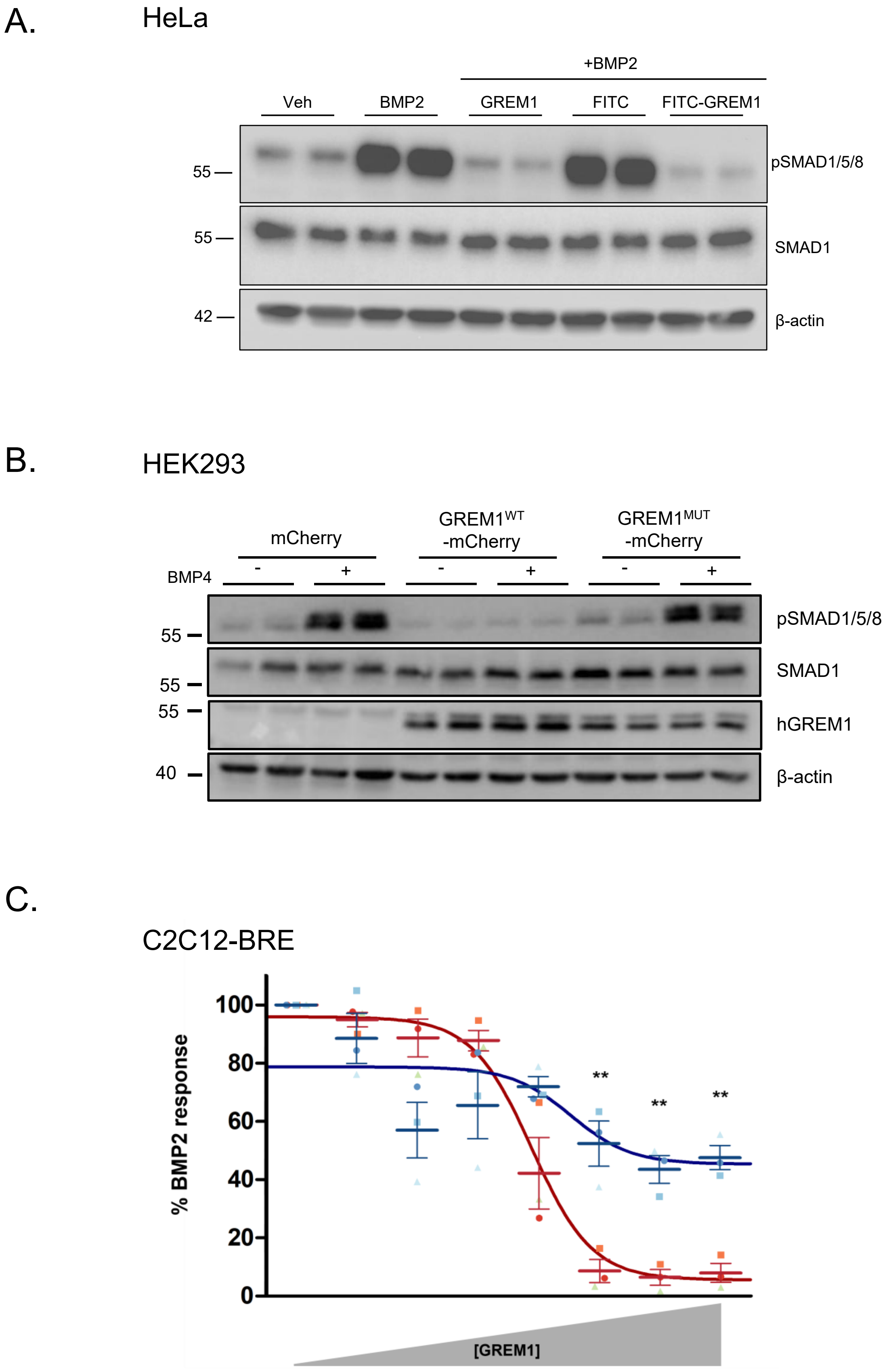

Supporting Figure 1. BMP2 and BMP4 activity is inhibited by GREM1-FITC and GREM1^{WT}-mCherry, but not GREM1^{MUT}-mCherry. A. HeLa cells were cultured in medium containing 1 % FBS overnight, followed by 3 h serum-free treatment. Cells were then treated for 60 min at 37 ° C with either vehicle (4 mM HCl and 0.1 % BSA) or 5 ng/mL rhBMP2 in the presence of either 25 ng/mL unconjugated rhGREM1, unconjugated FITC, or FITC-GREM1. Pre-incubation of FITC-GREM1 with vehicle or 5 ng/mL rhBMP2 was performed for 15 min at 37 ° C. Protein was then extracted and analysed by Western blotting using pSMAD1/5/8 and total SMAD1 antibodies. β-actin was used as loading control. Figure is representative of n=3 independent experiments. B. HEK293 cells were transfected with plasmids expressing mCherry, GREM1^{WT}-mCherry and GREM1^{MUT}-mCherry. After 24 h, cells were treated with either vehicle (4 mM HCl and 0.1 % BSA) or 5 ng/mL BMP4 for 1 h. Cell lysates were analyzed by Western blot using antibodies reactive to pSMAD1/5/8, total SMAD1, hGREM1 and mCherry. β-actin was used as a loading control. Data representative of n=3 independent experiments carried out in duplicate. C. C2C12 cells stably transfected with BMP response elements (C2C12-BREs, Inman ref) treated with increasing amounts of CM containing GREM1^{WT}-mCherry or GREM1^{MUT}-mCherry overnight (16 h) prior to lysis. Luciferase activity was assessed in 96-well plate format. Icons are representative of the average of duplicate parallel tests, carried out in triplicate wells. Luciferase response curves were generated using GraphPad Prism to determine half-maximal inhibitory values (IC_{50}). Data are plotted as mean ± SEM. One-way ANOVA followed by Bonferroni post-hoc test was used for statistical analysis. **, p<0.01.

Supporting Figure 1.
