# Supplementary material for: Identification of a novel GREMLIN1 uptake pathway in epithelial cells that requires BMP binding
Source: J Biol Chem. 2025 Sep 29;301(11):110780. doi: 10.1016/j.jbc.2025.110780 (PMC12597263; doi:10.1016/j.jbc.2025.110780)
Supplement: Supporting Figure S2 [file mmc3.pdf]

A.

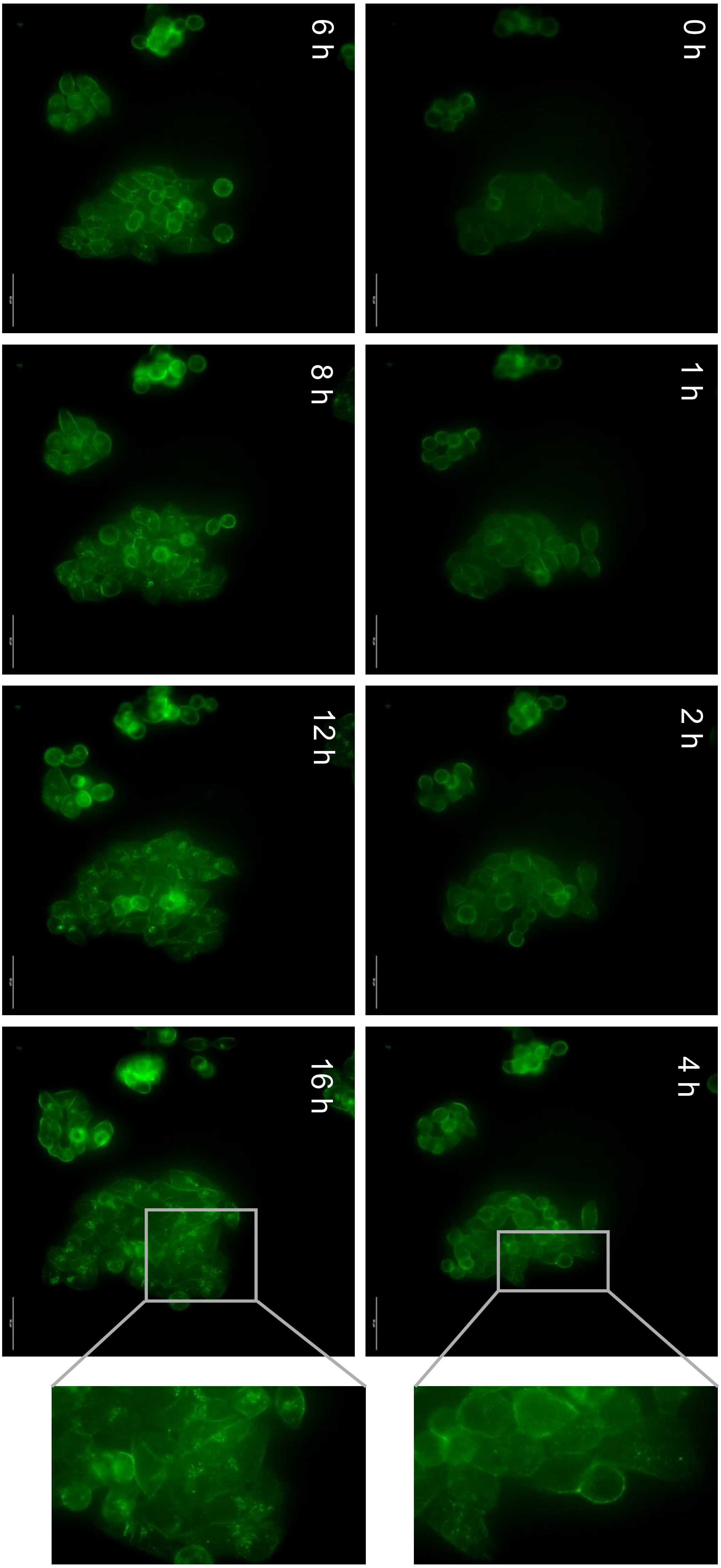

Supporting Figure 2.

B.

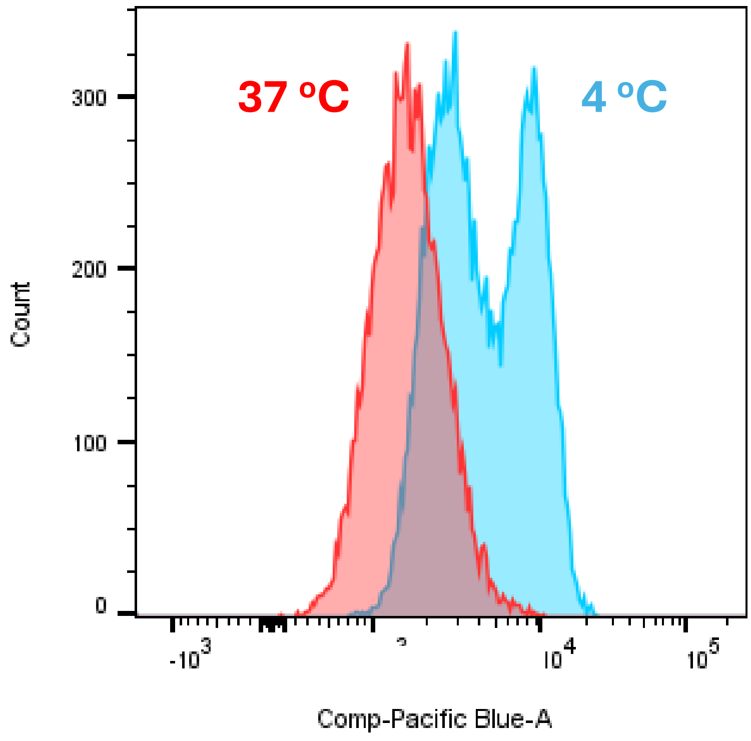

C.

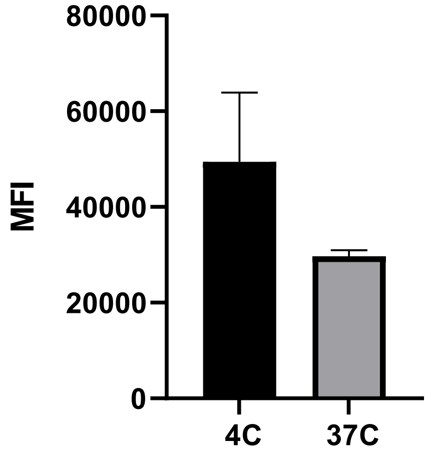

Supporting Figure 2. GREM1-FITC internalization in HCT116 cells. A. HCT116 cells were treated overnight with 1 µg/mL GREM1-FITC. Bright field and fluorescence “snapshot” images were captured every 30 min using a Nikon 6D live cell imaging microscope, and a live cell imaging video was compiled. Exported images at the indicated time-points demonstrate GREM1-FITC localization. The zoomed-in sections highlight the detailed localization of GREM1-FITC within the cells. Scale bar, 50 µm. B, C. HCT116 cells were incubated with biotinylated GREM1 at 4 °C and 37 °C overnight. Cells were stained with Streptavidin antibody and mean fluorescence intensity (MFI) quantified using flow cytometry. B. Flow histogram of Pacific Blue fluorescence intensity (x-axis) versus cell count (y-axis), showing a leftward shift for 37 °C v 4 °C samples, indicative of reduced streptavidin binding in this cell population. C. Quantitation of mean fluorescence intensity (MFI) for HCT cells incubated at 4 °C and 37 °C after incubation with GREM1-biotin and streptavidin staining. Data represent n=3 independent experiments.

Supporting Figure 2.
