# Supplementary material for: Identification of a novel GREMLIN1 uptake pathway in epithelial cells that requires BMP binding
Source: J Biol Chem. 2025 Sep 29;301(11):110780. doi: 10.1016/j.jbc.2025.110780 (PMC12597263; doi:10.1016/j.jbc.2025.110780)
Supplement: Supporting Figure S3 [file mmc4.pdf]

A. Mitochondria

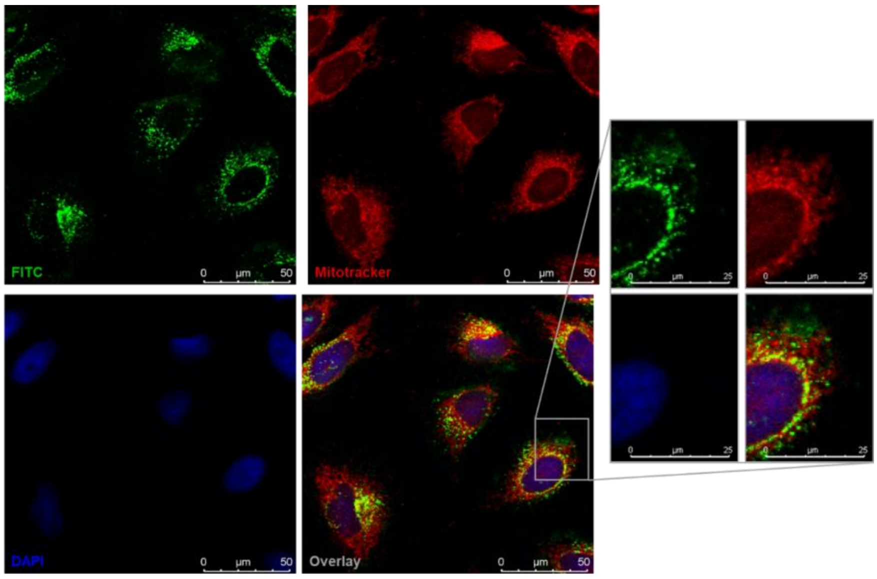

B. Endoplasmic Reticulum

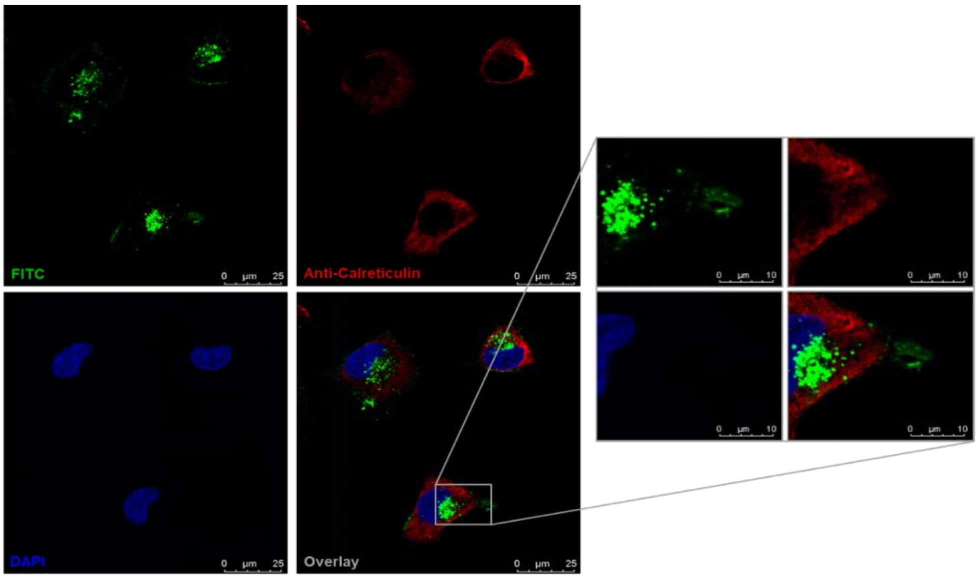

C. Golgi Apparatus

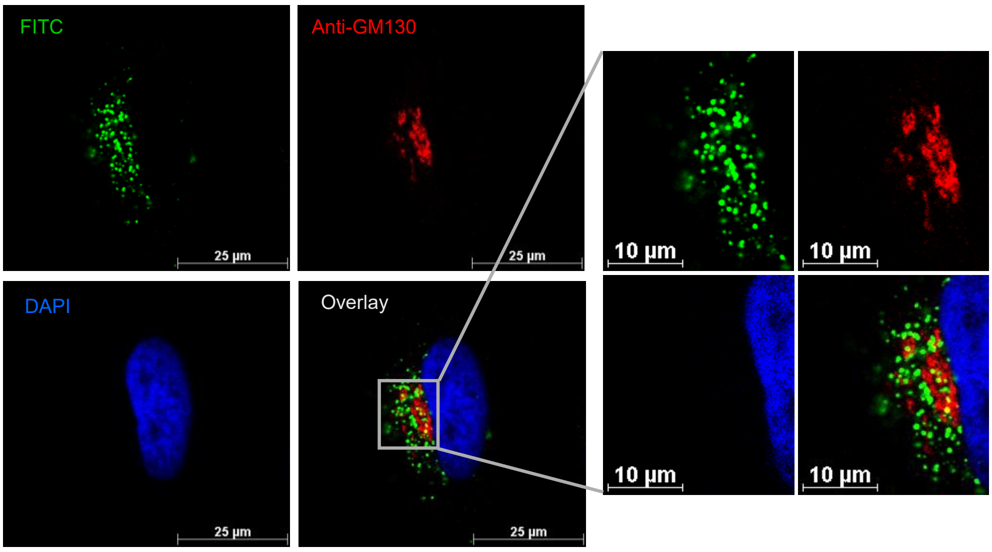

Supporting Figure 3. GREM1 does not localize to mitochondria, endoplasmic reticulum or Golgi apparatus. HeLa cells were seeded to a sparse density on Ibidi µ-Slides. After adherence, cells were treated with 1 µg/mL GREM1-FITC (green) overnight in complete medium before being stained with (A) 200 nM Mitotracker (red) to visualize mitochondria, (B) anti-Calreticulin (red) to visualize endoplasmic reticulum or (C) anti-GM130 (red) to visualize Golgi apparatus in serum-free growth medium for 30 min at 37 ° C. Cells were then stained with DAPI (blue) to visualize the nucleus. Slides were then images at 40 x magnification on the confocal microscope Leica SP5 as described in Methods. Scale bars represent 50 µm (A), 25 µm (B, C) or 25 µm (A), 10 µm (B, C) in the magnified panels. Data representative of n=3 independent experiments.

Supporting Figure 3.
