# Supplementary material for: Identification of a novel GREMLIN1 uptake pathway in epithelial cells that requires BMP binding
Source: J Biol Chem. 2025 Sep 29;301(11):110780. doi: 10.1016/j.jbc.2025.110780 (PMC12597263; doi:10.1016/j.jbc.2025.110780)
Supplement: Supporting Figure S4 [file mmc5.pdf]

A. Lysosomes

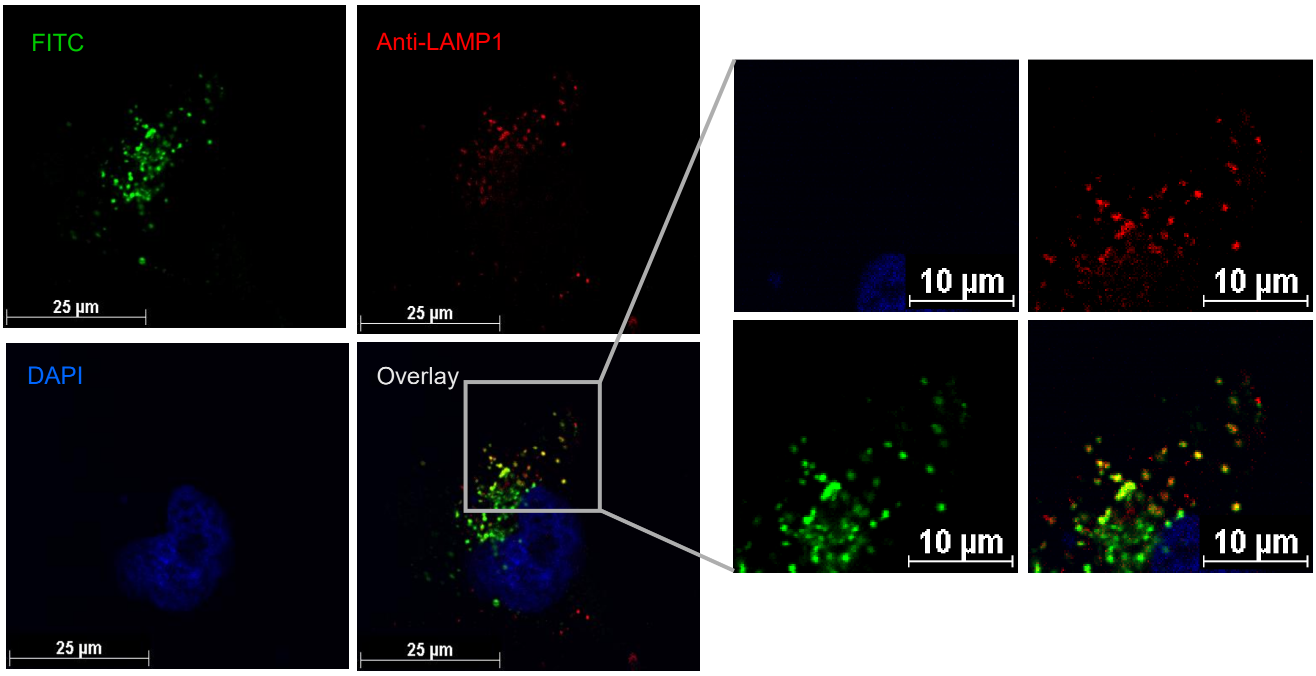

B. Recycling endosomes

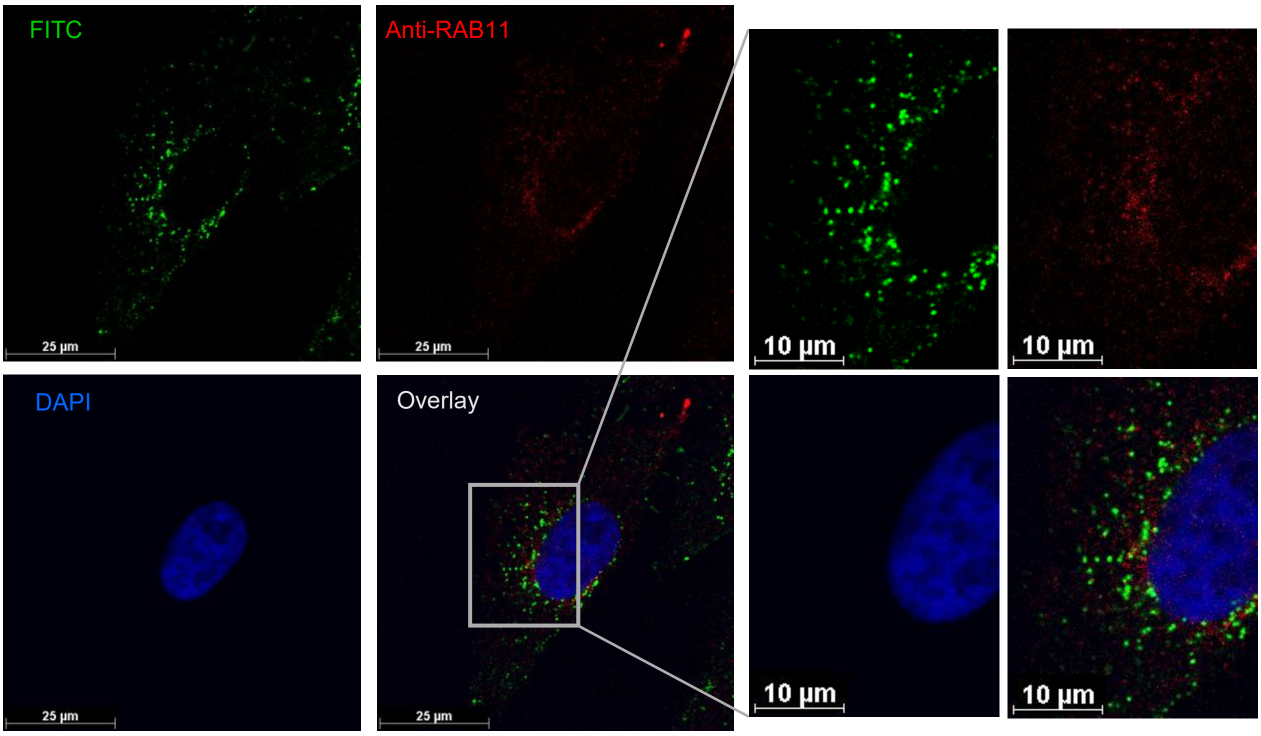

Supporting Figure 4. GREM1 partially localizes to lysosomes but not recycling endosomes. HeLa cells were treated with 0.5 µg/mL GREM1-FITC overnight in complete medium. Cells were then washed with PBS and fixed with 4 % PFA (w/v) before staining with (A) anti-LAMP-1 to visualize lysosomes (red) or (B) anti-RAB11 to stain for recycling endosomes, and DAPI (blue) to visualize nuclei. Slides were then imaged at 100 x magnification on a Leica SP5 confocal microscope. Scale bars represent 25 µm or 10 µm in magnified images. Data representative of n=3 independent experiments.

Supporting Figure 4.
