# Supplementary material for: Identification of a novel GREMLIN1 uptake pathway in epithelial cells that requires BMP binding
Source: J Biol Chem. 2025 Sep 29;301(11):110780. doi: 10.1016/j.jbc.2025.110780 (PMC12597263; doi:10.1016/j.jbc.2025.110780)
Supplement: Supporting Figure S5 [file mmc6.pdf]

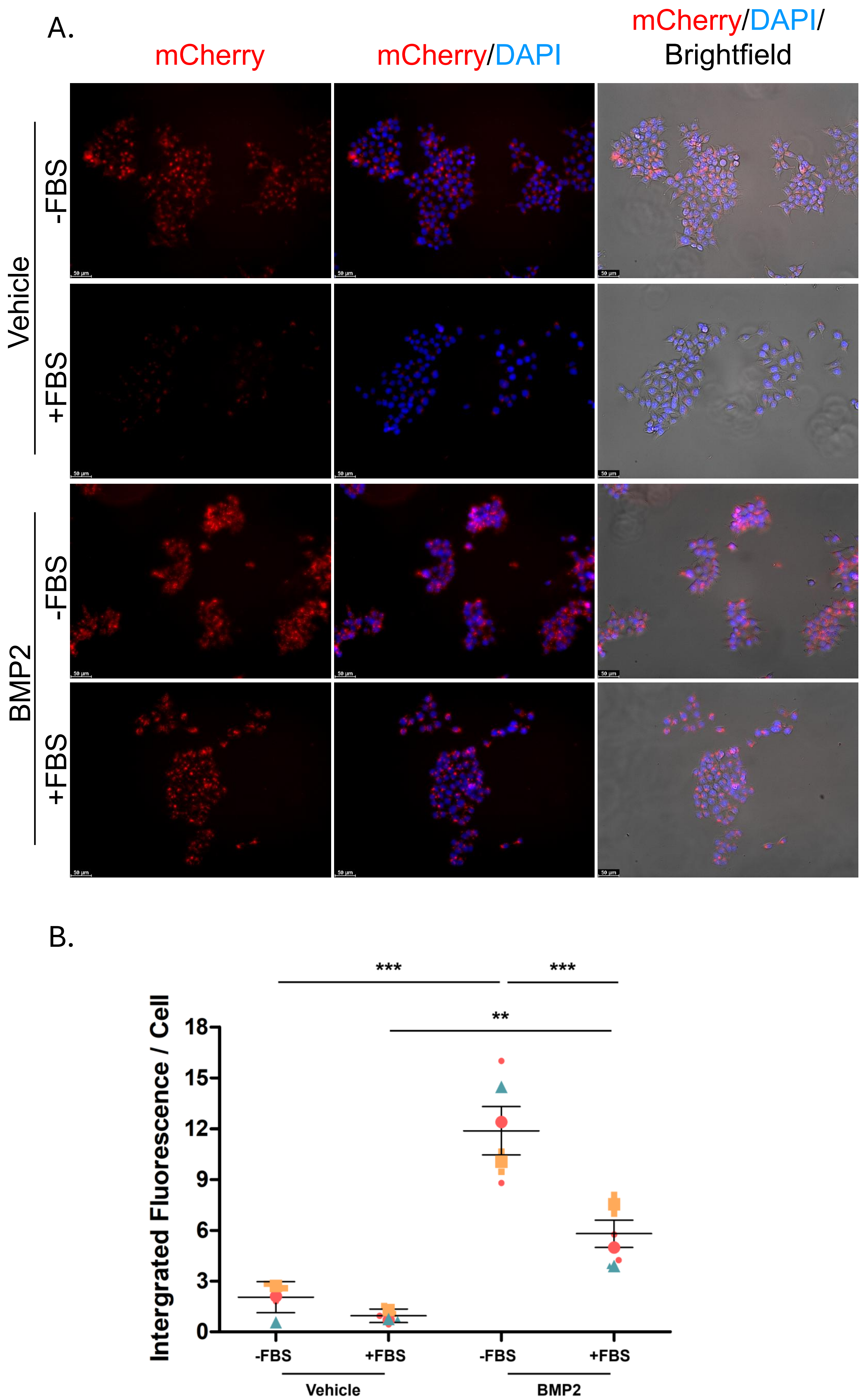

Supporting Figure 5. BMP2-stimulated GREM1 uptake requires FBS. A. Conditioned medium (CM) from HEK293 cells transfected with GREM1^{WT}-mCherry was added to HCT116 cells in the presence of vehicle (4 mM HCl and 0.1 % BSA) or 200 ng/mL BMP2 overnight (16 h) in either serum-free (-FBS) or 10 % FBS (+FBS)-containing medium. Cells were fixed and stained with DAPI before imaging using a Leica DMi8 microscope at 20 x magnification. B. Integrated fluorescence per cells was quantified by ImageJ followed by plotting using Graphpad Prism. Data are presented as mean ± SEM. Large icons represent the mean fluorescence intensity from three independent experiments (n=3). Small icons indicate the average of duplicate wells, with three images taken per well (except for the 3rd time experiment, which included only one well). Statistical analysis was determined using one-way ANOVA followed by Bonferroni post-hoc test. (**, $p<0.01$; ***, $p<0.001$)
